# Plasma p‐tau217 identifies cognitively normal older adults who will develop cognitive impairment in a 10‐year window

**DOI:** 10.1002/alz.14537

**Published:** 2025-02-26

**Authors:** Yara Yakoub, Fernando Gonzalez‐Ortiz, Nicholas J. Ashton, Christine Déry, Cherie Strikwerda‐Brown, Frédéric St‐Onge, Valentin Ourry, Michael Schöll, Maiya R. Geddes, Simon Ducharme, Maxime Montembeault, Pedro Rosa‐Neto, Jean‐Paul Soucy, John C. S. Breitner, Henrik Zetterberg, Kaj Blennow, Judes Poirier, Sylvia Villeneuve, PREVENT−AD Research Group

**Affiliations:** ^1^ Douglas Mental Health University Institute Centre for Studies on the Prevention of Alzheimer's Disease (StoP‐AD) Montreal Quebec Canada; ^2^ Department of Psychiatry and Neurochemistry Institute of Neuroscience and Physiology The Sahlgrenska Academy, University of Gothenburg Gothenburg Sweden; ^3^ Centre for Age‐Related Medicine Stavanger University Hospital Stavanger Norway; ^4^ King's College London Institute of Psychiatry, Psychology & Neuroscience Maurice Wohl Clinical Neuroscience Institute London UK; ^5^ Banner Alzheimer's Institute Phoenix Arizona USA; ^6^ School of Psychological Sciences The University of Western Australia Perth Western Australia Australia; ^7^ Department of Neurodegenerative Disease UCL Queen Square Institute of Neurology University College London London UK; ^8^ Montreal Neurological Institute McGill University Montreal Quebec Canada; ^9^ McGovern Institute for Brain Research Massachusetts Institute of Technology Cambridge Massachusetts USA; ^10^ McGill University Research Centre for Studies in Aging, McGill University Montreal Quebec Canada; ^11^ Department of Psychiatry McGill University Montreal Quebec Canada; ^12^ Clinical Neurochemistry Laboratory Sahlgrenska University Hospital Mölndal Sweden; ^13^ UK Dementia Research Institute at UCL London UK; ^14^ Hong Kong Center for Neurodegenerative Diseases Hong Kong Hong Kong; ^15^ UW Department of Medicine School of Medicine and Public Health Madison Wisconsin USA

**Keywords:** amyloid, CSF, MCI, PET, plasma, tau

## Abstract

**INTRODUCTION:**

We assessed the prognostic accuracy of plasma p‐tau217 in predicting the progression to mild cognitive impairment (MCI) in cognitively unimpaired (CU) individuals over a mean follow‐up of 5.65 years after plasma collection (range 1.01–10.47).

**METHODS:**

We included 215 participants from the PREVENT−AD cohort with plasma Aβ_42/40_ and p‐tau217, 159 with cerebrospinal fluid (CSF) Aβ_42/40_ and p‐tau217, and 155 with ^18^F‐NAV4694 and ^18^F‐flortaucipir PET scans. MCI progression was determined by multidisciplinary consensus among memory experts blind to biomarker and genetic information.

**RESULTS:**

Cox proportional hazard models indicated a greater progression rate in A+T+_plasma_ and A−T+_plasma_ compared to A−T−_plasma_ individuals (HR = 7.81 [95% CI = 3.92 to 15.59] and HR = 4.25 [1.60–11.31] respectively). Similar results were found with CSF (HR = 3.63 [1.72–7.70]) and PET (HR = 9.30 [3.67–23.55]).

**DISCUSSION:**

Plasma p‐tau217 is a prognostic marker for identifying individuals who will develop cognitive impairment within ten years.

**Highlights:**

- Elevated plasma p‐tau217 levels in CU individuals indicate future clinical progression.
- Adding plasma Aβ_42/40_ status to p‐tau markers did not improve the prediction to MCI.
- All individuals with abnormal tau PET measured in a temporal meta‐ROI progressed to MCI.

## 1 BACKGROUND

The core pathological hallmarks that define Alzheimer's disease (AD) are plaque‐forming aggregates of amyloid beta (Aβ) and neurofibrillary tangles of hyper‐phosphorylated tau (p‐tau). These proteins start to accumulate up to 20 years before the disease's clinical onset.^1^ They can be measured in vivo using positron emission tomography (PET); cerebrospinal fluid (CSF) assays; and more recently, blood‐based biomarkers.^2^, ^3^

PET and CSF biomarkers of Aβ and tau have been largely validated and can now be used to support AD diagnosis and to enroll AD patients in clinical trials. More recently, Aβ and tau PET have shown value in identifying cognitively unimpaired (CU) individuals at imminent risk (3–5 years) of developing mild cognitive impairment (MCI).^4^, ^5^ With the emergence of disease‐modifying therapies, it has become urgent to find low‐cost and widely available biomarkers that can identify CU individuals who will develop cognitive impairments. Prognostic information is therefore of critical value for informing treatment decisions, balancing risk/benefit, and establishing advance directives.

The field of plasma biomarkers has evolved dramatically over the past 5 years, with p‐tau217 plasma biomarkers emerging as one of the most promising at identifying individuals with AD pathology.^6^ However, imaging and fluid biomarkers of Aβ and tau reflect different biochemical pools of proteins; fluid biomarkers capture soluble and diffusible proteins, whereas PET images capture insoluble aggregates that are characteristic of the later stages of the disease.^7^ Consequently, it is hypothesized that Aβ and tau fluid biomarker abnormalities occur prior to PET abnormalities,^8^, ^9^, ^10^ and that PET abnormalities are more closely related to the development of cognitive impairments. Therefore, plasma p‐tau217 may not have the same prognostic value as tau PET in identifying CU individuals who will develop MCI.

In a longitudinal study spanning > 10 years, we assessed the prognostic value of novel plasma p‐tau217 assays in predicting progression from CU to MCI. The prognostic value of plasma markers was then compared to the prognostic value of CSF p‐tau217 and PET biomarkers. MCI classification was performed blind to genetic and pathological biomarkers.

## 2 METHODS

We included 215 participants from the Presymptomatic Evaluation of Experimental or Novel Treatments for Alzheimer Disease (PREVENT‐AD) cohort, a longitudinal observational study of individuals with a first‐degree family history of AD (Figure S1 in supporting information). Longitudinal data collected between 2011 and 2023 and available plasma (Aβ_42/40_ and p‐tau217) biomarkers, 159 participants with available CSF p‐tau217 and Aβ_42/40_ values, and 155 with both Aβ and tau PET available (Methods S1 in supporting information). Plasma and CSF measurements were collected from the beginning of the study while PET measurements started in 2017. Plasma and CSF time points closest to the PET scans were selected for this study (see Methods S1 and Figure S2 in supporting information). For individuals who did not have PET scans, the plasma sample closest to 2017 was taken. Plasma, CSF, and PET biomarkers were not necessarily collected on the same day. Participants included in this study were CU based on an extensive neuropsychological evaluation at the time of the biomarker assessment and had a minimum of 1 year of cognitive follow‐up thereafter. To ease comparison among biomarkers, a subsample of 93 participants with all biomarker modalities were included in a supplementary analysis. The demographic and clinical information of plasma, CSF, and PET full samples can be found in Table 1. The characteristics of the corresponding AT groups in plasma, CSF, and PET can be found in Tables S1–S3 in supporting information. The amyloid/tau (AT) group demographic characteristics of the subsample of 93 participants with all biomarkers can be found in Tables S4–S6 in supporting information. Written informed consent was obtained from all participants, and all research procedures were approved by the institutional review board at McGill University and complied with the ethical principles of the Declaration of Helsinki. A detailed description of the PREVENT‐AD cohort is available elsewhere.^11^

**TABLE 1:** Sample demographics.

Demographics	Plasma (*n* = 215)	CSF (*n* = 159)	PET (*n* = 155)	Group differences
**Age at baseline, years**	63.19 (4.85)	62.92 (4.80)	63.70 (4.62)	*P* = 0.14
**Age at biomarker classification, years** ^a^	65.16 (5.28)	64.75 (5.26)	67.64 (5.01)	*P* < 0.001^a^, ^b^
**Sex, *F* (%)**	157 (73)	113 (71)	111 (72)	*P* = 0.87
**Education, years**	15.29 (3.25)	15.14 (3.17)	15.33 (3.25)	*P* = 0.78
***APOE* ε4 carriers, *n* (%)**	88 (41)	62 (39)	62 (40)	*P* = 0.96
**Global amyloid SUVR**	NA	NA	1.31(0.30)	NA
**Temporal meta‐ROI SUVR**	NA	NA	1.16 (0.11)	NA
**Aβ_42/40_**	0.09 (0.01)	0.09 (0.02)	NA	NA
**p‐tau217** **(pg/ml)**	2.63 (1.56)	251.70 (173.65)	NA	NA
**MoCA score/30**	28.11 (1.57)	28.05 (1.58)	28.14 (1.51)	0.89
**RBANS global score**	101.57 (9.85)	101.10 (9.80)	102.54 (10.12)	0.22
**MMSE score/30**	28.81 (1.24)	28.80 (1.27)	28.83 (1.25)	0.98

*Note*: Represents the characteristics of plasma, CSF, and PET full sample and their corresponding AT groups. Data presented as mean (standard deviation), except for categorical variables where the count and percentage are presented. Fisher or Kruskal–Wallis test was performed between the groups and *P* values are reported in the right column. If a significant group difference (*P* < 0.05) was found post hoc tests are performed. Age at baseline and at biomarker measurement are presented; MoCA scores were collected at entry into the program; RBANS values are shown at baseline; MMSE scores selected closest to the biomarker measurement. MoCA score was missing for one participant in the plasma and CSF sample. MMSE scores were available for a subset of participants (*n* = 188 for plasma sample, *n* = 136 for CSF sample, and *n* = 154 for PET sample). Abbreviations: *APOE*, apolipoprotein E; AT, amyloid/tau; CSF, cerebrospinal fluid; F, Female; MMSE, Mini‐Mental State Examination; MoCA, Montreal Cognitive Assessment; PET, positron emission tomography; RBANS, Repeatable Battery for the Assessment of Neuropsychological Status; SUVR, standardized uptake value ratio.

^a^ Difference between plasma and PET.

^b^ Difference between CSF and PET.

### 2.1 Biomarker measurements and AT classification

Plasma and CSF p‐tau217 were measured using an in‐house Simoa platform developed at the Clinical Neurochemistry Laboratory, University of Gothenburg.^12^ Plasma Aβ_40_ and Aβ_42_ concentrations were measured using ultrasensitive immunoprecipitation coupled with mass spectrometry (IP‐MS) technique using a KingFisher Flex Purification System (Thermo Fisher Scientific) at the Clinical Neurochemistry Laboratory.^13^ CSF Aβ_40_ and Aβ_42_ were analyzed using LUMIPULSE G‐automated immunoassay (Fujirebio, Ghent, Belgium).^14^ Tau PET scans were performed using ^18^F‐flortaucipir and Aβ PET scans using ^18^F‐NAV4694.^4^

**RESEARCH IN CONTEXT**

1. **Systematic review**: We searched PubMed for plasma, CSF, and PET biomarkers of Alzheimer's disease (AD). Amyloid (A) and phosphorylated‐tau (p‐tau, T) blood biomarkers have been recently added to the revised criteria for diagnosis and staging of AD. There is limited evidence on whether plasma biomarkers can identify cognitively unimpaired (CU) who will develop mild cognitive impairment (MCI). In this prospective study, we evaluated the prognostic accuracy of amyloid and p‐tau plasma biomarkers in predicting MCI progression compared to CSF and PET biomarkers.
2. **Interpretation**: Our study provided strong evidence on the prognostic utility of plasma p‐tau217 in predicting future clinical progression. Amyloid and tau positive individuals in plasma showed higher risk for future cognitive impairment, similar to PET and CSF. Interestingly, amyloid markers did not improve the predictivity of the tau markers, which can be explained by the fact that almost all T+ participants were also A+.
3. **Future directions**: Future studies in diverse populations and community settings are needed to validate our findings.

Aβ PET images using ^18^F‐NAV4694 as tracer were captured 40 to 70 minutes after injecting a targeted dose of 220 MBq (6 mCi). Tau PET images using ^18^F‐flortaucipir as tracer were obtained 80 to 100 minutes after injection with a targeted dose of 370 MBq (10 mCi). We acquired six frames of 5 minutes for NAV and four frames of 5 minutes for FTP. An in‐house pipeline (https://github.com/villeneuvelab/vlpp) was used for the preprocessing of all PET scans (see supporting information for more details). For the main analyses, Aβ was quantified in a global index^15^, ^16^ including temporal, parietal, and frontal regions of interest (ROIs), while tau was quantified in a temporal meta‐ROI (see supporting information for more details).^17^ The analyses were repeated when tau was solely quantified in the entorhinal cortex, a region with early tau accumulation.^18^, ^19^

For the binary analyses, plasma and CSF p‐tau217 levels were set at 2 standard deviation (SD) above the mean of Aβ PET–negative individuals who were CU from the PREVENT‐AD cohort (plasma p‐tau217: 3.98 pg/mL; CSF p‐tau217: 400.19 pg/mL).^20^ For tau positivity, we used a pre‐established threshold of 1.29 standardized uptake value ratio (SUVR) in a temporal meta‐ROI, that was also based on 2 SD above the mean of CU Aβ PET–negative individuals from our cohort.^17^ Using the same method, the entorhinal tau PET threshold corresponded to a SUVR of 1.22. We used the pre‐established thresholds of 0.09 for plasma Aβ_42/40_ and 0.072 for CSF Aβ_42/40_.^13^, ^14^ Aβ PET positivity threshold was SUVR of 1.26 and was determined as the midpoint between the liberal and the conservative threshold.^4^ The liberal threshold (SUVR 1.18) was calculated as 2 SD above the mean of 11 young individuals (< 40 years old) who underwent the same PET protocol, while the conservative threshold (SUVR 1.33) was established using Gaussian mixture modeling. While we are using pre‐established methods to define our thresholds for the main analyses to increase generalizability of the findings, Tables S7–S12 in supporting information further show the sensitivity, specificity, negative predictive value, and positive predictive value of all other possible thresholds, derived using progression status as the outcome variable.

### 2.2 Outcomes

MCI classification was determined in a reserch setting based on a multidisciplinary consensus meeting comprising dementia specialist neuropsychologists (SV and MM) and physicians (SD and MG). Individuals with a cognitive performance deviating by more than 1.0 SD from demographically stratified norms on at least one of the five composite subscale scores (immediate memory, delayed memory, attention, visuospatial ability, and language) of the Repeatable Battery for the Assessment of Neuropsychological Status (RBANS), or on two subset scores on the Rey Auditory Verbal Learning Test (RAVLT) were revised in consensus. When reviewed in consensus meeting, cognitive data at all available time points were examined. In addition to an objective impairment on the RBANS or the RAVLT, to be classified as MCI, participants needed to have a subjective memory complaint^21^ and/or have an objective decline witnessed over multiple cognitive follow‐ups. All MCI classification was performed blind to plasma, CSF, PET, magnetic resonance imaging (MRI), and apolipoprotein E (*APOE*) genotype information.^4^ Most MCI participants were later evaluated in a clinical setting and the most recent available information related to the severity of the cognitive impairment is reported in Figure S3 in supporting information. This information is not treated as a main outcome as it was obtained unblinded to *APOE* genotype and AD biomarker status.

In secondary analyses, we examined the longitudinal cognitive trajectories of individuals using the total composite score of the RBANS as an outcome. The RBANS is administered to PREVENT‐AD participants throughout the entire study follow‐up. These secondary analyses took advantage of all cognitive time points, including cognitive evaluations prior to and after the biomarker classification, except for virtual evaluations performed during the COVID‐19 pandemic.

### 2.3 Statistical analyses

Demographic and clinical variables by biomarker and by AT classification were compared using Kruskal–Wallis tests followed by a Dunn post hoc test for continuous variables and Fisher exact tests for categorical variables. Cox proportional hazard models were used to assess the risk of MCI progression by AT group and linear mixed models were used to assess longitudinal cognitive changes across the biomarker groups with the addition of age at the biomarker classification, sex, and years of education as covariates. We then tested the cognitive performance over time across the different biomarker groups using linear mixed‐effects models. Change in the age‐adjusted RBANS total score over time was used as an outcome variable with time and biomarker group as interaction terms, with a random slope and intercept for time per subject. Each model was adjusted for sex and education as potential confounders (e.g., [RBANS ∼ time × AT biomarker group + sex + years of education + (time | subject)]) using the A−T− group as the reference group. Spearman rank test and Cohen kappa were used to evaluate the association between the biomarkers. Receiver operating characteristic (ROC) analyses were used to test the performance of plasma, CSF, and PET biomarkers as continuous variables in predicting the progression to MCI. The resulting areas under the curve (AUCs) were also computed to assess the biomarker accuracy in distinguishing between CU who remained CU during the study length versus those who progressed to MCI. These last analyses were done using Aβ and tau in separate models and when combined, to assess the additive value of having Aβ markers in addition to the tau biomarkers. Model fits were compared using a Vuong test. Two‐sided *P* values < 0.05 were deemed significant. The analyses were performed using the R programming language.

## 3 RESULTS

### 3.1 Participants

A total of 228 participants were included in the study, of whom 62 (27%) developed MCI. Across all participants, the mean duration of cognitive follow‐up including time points prior to the biomarker assessment was 7.7 years (SD = 1.93, range 1.39–10.49 years). The mean age of the full sample at baseline was 63 years, 72% were female, and 39% were *APOE* ε4 carriers. The demographic and clinical profiles of the plasma (*n* = 215), CSF (*n* = 159), and PET (*n* = 155) samples is presented in Table 1; the breakdown by AT biomarker groups can be found in Tables S1–S3. The mean cognitive follow‐up was 5.65 years (SD = 1.45, range 1.01–10.47) after plasma classification, 5.57 years (SD = 1.48, range 1.00–10.00) after CSF classification, and 4.18 years (SD = 1.49, range 1.02–6.07) after PET classification. The full cognitive follow‐up length, which for most participants included evaluations prior to the biomarker assessment, was 7.63 years (SD = 1.94, range: 1.00–10.47) for the plasma sample, 7.59 years (SD = 1.89, range: 1.89–10.47) for the CSF sample, and 8.11 (SD = 1.76, range: 2.99–10.49) for the PET sample.

### 3.2 AT classification across biomarkers

Using Aβ_42/40_ and p‐tau217 plasma biomarkers to classify participants, 10% (21/215) were classified as A+T+, 28% (60/215) as A+T−, 3% (7/215) as A−T+, and 59% (127/215) as A−T−. Using Aβ_42/40_ and p‐tau217 CSF biomarkers, 11% (18/159) were classified as A+T+, 3% (5/159) as A+T−, 2% (3/159) as A−T+, and 84% (133/159) as A−T−. Finally, using PET biomarkers, 5% (8/155) were classified as A+T+, 29% (45/155) as A+T−, 1% (1/155) as A−T+, and 65% (101/155) as A−T−. While the proportion of CU A+T+ participants classified with PET was low, it is similar to what has been found in other PET studies^4^, ^5^ and can probably be explained by that fact that most individuals positive on both PET markers have cognitive impairments. In line with previous studies, using the entorhinal cortex rather than a temporal meta‐ROI to classify tau positivity slightly increased the number of A+T+ participants.^4^, ^5^

### 3.3 Rate of progression from CU to MCI across AT groups when defined using fluid versus neuroimaging biomarkers

**FIGURE 1:**
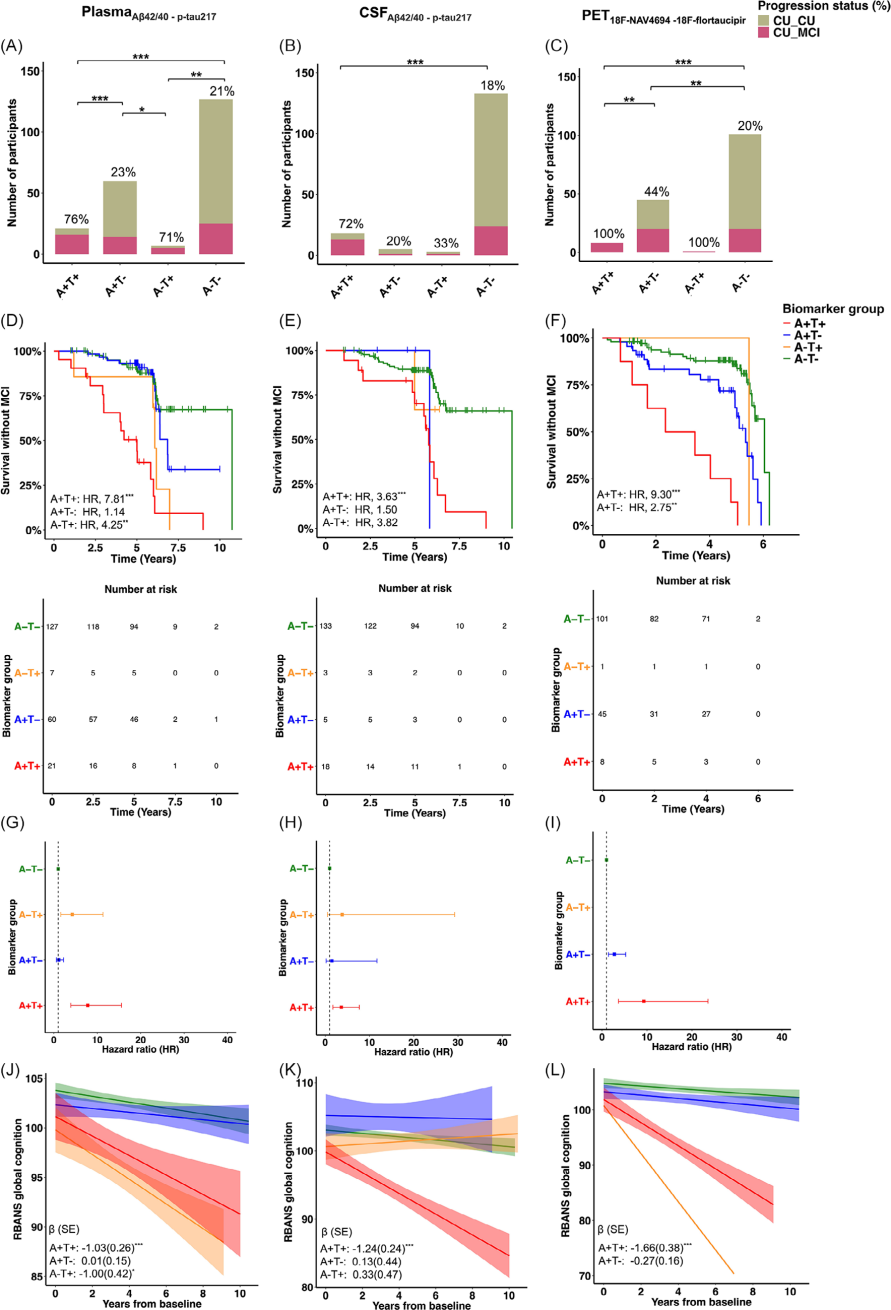
Clinical progression to MCI across plasma, CSF, and PET AT biomarker groups. Bar graphs represent the proportion of participants who developed MCI across (A) plasma Aβ_42/40_ and p‐tau217, (B) CSF Aβ_42/40_ and p‐tau217, and (C) PET biomarker profiles measured with ^18^F‐NAV4694 and ^18^F‐flortaucipir. Survival curves reflecting the progression to MCI across (D) plasma, (E) CSF, and (F) PET biomarker groups. The vertical ticks on the curves refer to censored participants, that is, the lack of follow‐up of the individuals. G–I, Forest plots showing HR and 95% confidence intervals from the survival analyses. Linear mixed effects models show the total RBANS cognitive score over time across (J) plasma, (K) CSF, and (L) PET biomarker profiles. The linear mixed effects models analyses included annual cognitive data before and after plasma, CSF, and PET measures. Models included age at biomarker measurement, sex, and years of education as covariates. Tau PET positivity was determined using temporal meta‐ROI. The A−T− group was used as reference. The A−T+ in the PET (*n* = 1) biomarker group is displayed for visualization purposes but was not included in the statistical analyses. The exact number of participants per group can be found in the first column of the lower part of panel (D), (E), and (F; number at risk at time 0). ^*^ *P *< 0.05, ^**^P < 0.01, ^***^p < 0.001. Aβ, amyloid beta; AT, amyloid, tau; CSF, cerebrospinal fluid; CU_CU, cognitively unimpaired older adults at the time of the biomarker measurement and remained cognitively unimpaired during follow‐up; CU_MCI, cognitively unimpaired older adults at the time of the biomarker measurement, who progressed to mild cognitive impairment during follow‐up; HR, hazard ratio; MCI, mild cognitive impairment; PET, positron emission tomography; p‐tau, phosphorylated tau; RBANS, Repeatable Battery for the Assessment of Neuropsychological Status; SE, standard error.

Seventy‐six percent (16/21) of the A+T+_plasma_ group developed MCI compared to 23% (14/60) in the A+T−_plasma_ group, 71% (5/7) in the A−T+_plasma_ group, and 21% (25/127) in the A−T− _plasma_ group (Figure 1A). The proportion of CU developing MCI was higher in the A+T+_plasma_ group compared to A−T−_plasma_ and A+T− _plasma_ groups (Fisher exact *P* < 0.001). While the A−T+_plasma_ group was small it nevertheless included a higher proportion of participants who developed MCI compared to the A+T−_plasma_ and A−T−_plasma_ groups (Fisher exact *P* = 0.02, *P* = 0.006, respectively). When the groups were classified using CSF, 72% (13/18) of the A+T+_CSF_, 20% (1/5) of the A+T−_CSF_ group, 33% (1/3) of the A−T+, and 18% (24/133) of the A−T−_CSF_ group developed MCI (Figure 1B). An increased CU to MCI progression rate was found in the A+T+_CSF_ group compared to A−T−_CSF_, but no differences were found compared to the A+T−_CSF_ group (Fisher exact *P* < 0.001 and *P* = 0.06, respectively). In the PET groups, 100% (8/8) of A+T+_PET_ biomarker group, 44% (20/45) of the A+T−_PET_ group, 100% (1/1) of the A−T+, and 20% (20/101) of the A−T−_PET_ group progressed to MCI (Figure 1C). The A+T+_PET_ group was associated with increased progression to MCI compared to A−T−_PET_ and A+T−_PET_ (Fisher exact *P* < 0.001, *P* = 0.005, respectively). We also found differences between the A+T−_PET_ and A−T−_PET_ groups (Fisher exact *P* = 0.005). One hundred twenty‐eight of these 155 PET participants were included in a previously published study,^4^ which showed that 55% of A+T+_PET_, 9% of A+T−_PET_, and 10% of A−T−_PET_ participants developed MCI when followed for a mean of 3.16 years after the AT classification. Now with an additional 2.4 years of cognitive follow‐up, 100% of these A+T+_PET_, 44% of these A+T−_PET_, and 20% of these A−T−_PET_ have developed MCI.

Results were replicated when restricting the sample size to the participants with all biomarker measurements, with all A+T+ groups showing a higher percentage of progression compared to their respective A−T− groups (the % of CU who progressed to MCI was 88% with plasma, 86% with CSF, and 100% with PET in the A+T+ compared to 14%–20% in the A−T− groups, Figure S4 in supporting information). Using the entorhinal cortex rather than the temporal meta‐ROI increased the number of T+_PET_ participants by > 50% (20 instead of 9), and yields results more similar to the CSF and plasma classification with 91% of A+T+ progressors in the restricted sample, 30% of A+T−, 50% of A−T−, and 20% of A−T−, results that were almost identical in the full sample (Figure S5 in supporting information).

Cox proportional hazard models showed a higher risk of progression from CU to MCI among the A+T+_plasma_ and A−T+_plasma_ (hazard ratio [HR] = 7.81, *P* < 0.001, 95% confidence interval [CI] = 3.92 to 15.59; HR = 4.25; *P* = 0.004, 95% CI = 1.60 to 11.31; model concordance value [model fit] = 0.72; standard error [SE] = 0.04; Figure 1D,G) compared to the A−T−_plasma_ (reference) group. There were no differences between A+T−_plasma_ and A−T−_plasma_ group (HR = 1.14, *P* = 0.69, 95% CI = 0.58 to 2.24). In the CSF sample, we found an increase in the risk among A+T+_CSF_ (HR = 3.63, *P* < 0.001, 95% CI = 1.72 to 7.70; model concordance value = 0.68; SE = 0.06; Figure 1E,H) compared to the A−T−_CSF_ group. No differences were found comparing the reference group to either A+T−_CSF_ or A−T+_CSF_ (HR = 1.50; *P* = 0.69, 95% CI = 0.19 to 11.69; HR = 3.82, *P* = 0.20, 95% CI = 0.50 to 29.24, respectively). Finally, A+T+_PET_ and A+T−_PET_ participants exhibited a higher risk of MCI progression compared to A−T−_PET_ (HR = 9.30, *P* < 0.001, 95% CI = 3.67 to 23.55; HR = 2.75, *P* = 0.002, 95% CI = 1.43 to 5.27; model concordance value = 0.73; SE = 0.04; Figure 1F,I). The A−T+ group was not included in the analyses given that only one participant was classified as A−T+_PET_; this participant nevertheless developed MCI during the study follow‐up. Results were replicated in the subsample of 93 participants with all biomarkers (Figure S4).

Finally, while not blind to biomarker status, all A+T+ MCI later seen in a clinical setting were classified as having MCI due to AD and ≈ 33% of them have now progressed to dementia while none of the A−T− received a diagnosis of AD, none progressed to dementia, and ≈ 25% were considered to have only subjective cognitive decline (Figure S3).

### 3.4 Cognitive trajectories

We also investigated the longitudinal cognitive performance of participants within the AT biomarker groups using plasma, CSF, and PET biomarkers while taking advantage of all cognitive time points, including the ones before the biomarkers’ classifications, when available. The A+T+_plasma_ and A−T+_plasma_ groups demonstrated steeper cognitive declines compared to A−T−_plasma_ (reference) group (*β* = −1.03, *P* < 0.001, SE = 0.26, 95% CI = −1.53 to −0.52; *β* = –1.00, *P* = 0.02, SE = 0.42, 95% CI = −1.83 to −0.18; *R* ^2^ = 0.12; Figure 1J), while no differences were observed between the A−T−_plasma_ and A+T−_plasma_ groups (*β* = 0.01, *P* = 0.96, SE = 0.15, 95% CI = −0.29 to 0.31). Using CSF to classify participants, the A+T+_CSF_ group showed a faster decline over time compared to A−T−_CSF_ (*β* = −1.24, *P* < 0.001, SE = 0.24, 95% CI = −1.72 to −0.77; *R* ^2^ = 0.11; Figure 1K), but no differences were found between the reference group and A+T−_CSF_ or the A−T+_CSF_ groups (*β* = 0.13, *P* = 0.77, SE = 0.44, 95% CI = −0.74 to 1.00; *β* = 0.33, *P* = 0.48, SE = 0.47, 95% CI = −0.58 to 1.24). When the groups were classified based on PET, the A+T+_PET_ group demonstrated a steeper cognitive decline compared to A−T−_PET_ (*β* = −1.66, *P* < 0.001, SE = 0.38, 95% CI = −2.40 to −0.91; Figure 1L). The A+T−_PET_ group demonstrated no differences compared to A−T−_PET_ group (*β* = −0.27, *P* = 0.10, SE = 0.16, 95% CI = −0.59 to 0.05). Identical results were found in the subsample of 93 participants (see Figure S4J–L for more details).

### 3.5 Concordance between different biomarkers modalities

**FIGURE 2:**
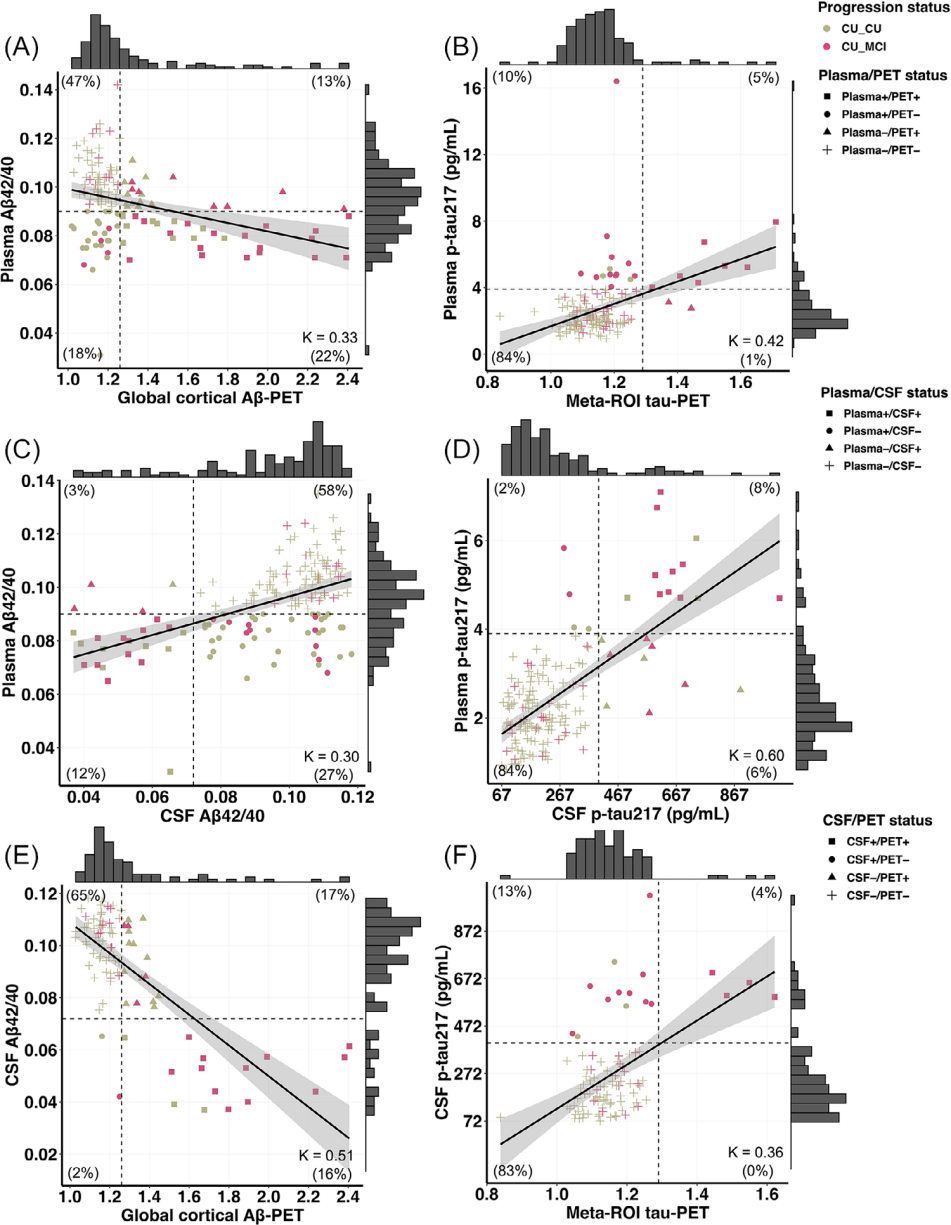
Scatterplots reflecting concordance status among plasma, CSF, and PET Aβ and tau biomarkers. Correlation plots of (A) plasma Aβ42/40 versus Aβ PET biomarkers; (B) plasma p‐tau217 versus tau PET biomarkers; (C) plasma Aβ42/40 versus CSF Aβ42/40 biomarkers; (D) plasma p‐tau217 versus CSF p‐tau217 biomarkers; (E) CSF Aβ42/40 versus Aβ PET biomarkers; (F) CSF p‐tau217 versus tau PET biomarkers. Colors indicate participants’ cognitive status and symbols indicate Aβ/tau positive or negative status. Vertical and horizontal dashed lines correspond to plasma, PET, and CSF biomarker cutoff values, respectively. Cutoff values were 0.09 for plasma Aβ42/40; 0.072 for CSF Aβ42/40; 1.26 SUVR for Aβ PET; 3.98 pg/mL for plasma p‐tau217; 400.19 pg/mL for CSF p‐tau217; and 1.29 SUVR for temporal meta‐ROI tau PET. *n* = 143 participants had both plasma and PET measurements, *n* = 158 had both plasma and CSF measurements, and *n* = 93 had both CSF and PET measurements. Aβ, amyloid beta; CSF, cerebrospinal fluid; CU_CU, cognitively unimpaired older adults at the time of the biomarker measurement and remained cognitively unimpaired during follow‐up; CU_MCI, cognitively unimpaired older adults at the time of the biomarker measurement, who progressed to mild cognitive impairment during follow‐up; PET, positron emission tomography; p‐tau, phosphorylated tau; SUVR, standardized uptake value ratio

We found weak correlation between plasma Aβ_42/40_ and Aβ PET (*r* = −0.35, *P* < 0.001, Figure 2A), and between plasma p‐tau217 and tau PET (*r* = 0.38, *P* < 0.001, Figure 2B), but moderate correlations between plasma and CSF Aβ_42/40_ (*r* = 0.48, *P* < 0.001, Figure 2C) and plasma and CSF p‐tau217 (*r* = 0.51, *P* < 0.001, Figure 2D). Similarly, CSF Aβ_42/40_ and CSF p‐tau217 showed weak to moderate correlation with both Aβ and tau PET (*r* = −0.58; *r* = 0.40; *P* < 0.001, respectively, Figure 2E,F). When evaluating concordance using Aβ and tau status as categorical variables with Cohen kappa, we found weak agreement between plasma Aβ_42/40_ and Aβ PET (kappa = 0.33, 95% CI = 0.17 to 0.49), and between plasma p‐tau217 and tau PET (kappa = 0.42, 95% CI = 0.19 to 0.64). The agreement was also weak between plasma and CSF Aβ_42/40_ (kappa = 0.30, 95% CI = 0.16 to 0.43), but moderate to strong agreement for plasma and CSF p‐tau217 (kappa = 0.60, 95% CI = 0.41 to 0.80). For CSF and PET Aβ and tau status, the agreement was moderate for Aβ, but weak for tau (kappa = 0.51, 95% CI = 0.32 to 0.69; kappa = 0.36, 95% CI = 0.10 to 0.62, respectively). See Figure S6 in supporting information for the concordance when stratified by positive/negative status and for the percentage of participants who progressed to MCI by biomarker status.

### 3.6 Direct comparisons between fluid and imaging biomarkers

**FIGURE 3:**
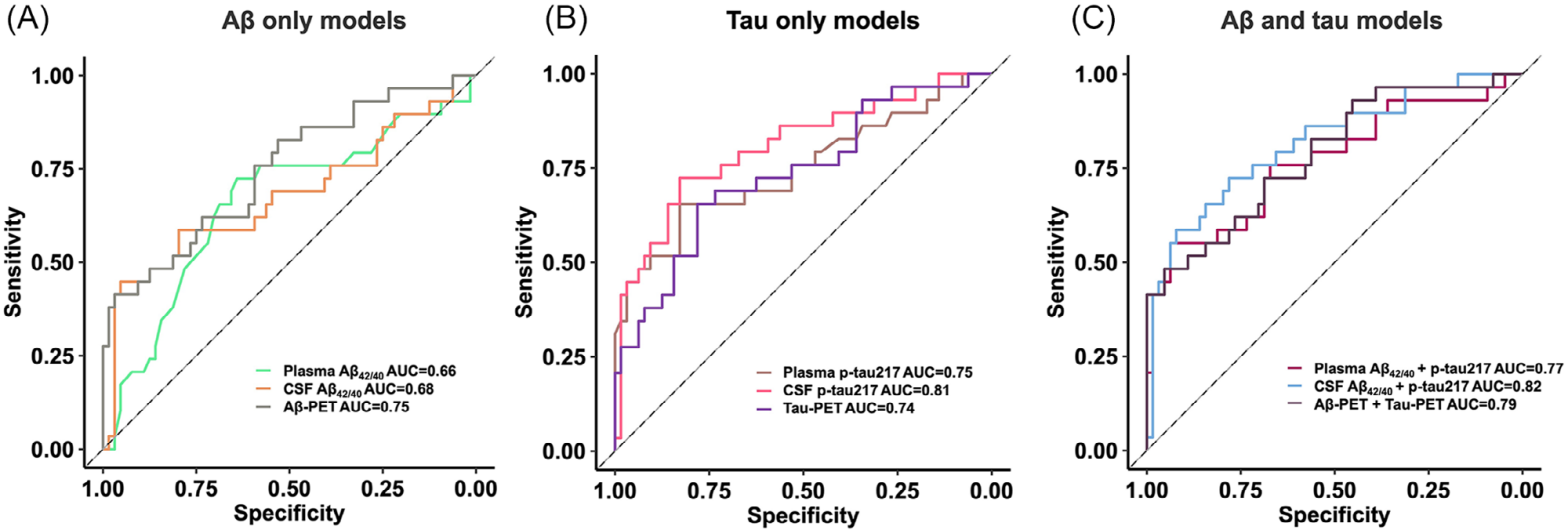
Discriminative accuracy of plasma, CSF, and PET biomarkers for identifying individuals who will progress to MCI. ROC curves and corresponding AUC showing the discriminative ability of (A) individual plasma, CSF, and PET Aβ models; (B) individual plasma, CSF, and PET tau models; and (C) models combining plasma Aβ_42/40_, CSF Aβ_42/40_, and Aβ PET biomarkers with plasma p‐tau217, CSF p‐tau217, and tau PET in distinguishing between individuals who remained cognitively normal versus those who developed MCI. Aβ, amyloid beta; AUC, area under the curve; CSF, cerebrospinal fluid; MCI, mild cognitive impairment; PET, positron emission tomography; ROC, receiver operating characteristic

Aβ, tau, and the combination of tau + Aβ models were comparable using the Vuong test. Comparing the performance of Aβ models, Aβ PET showed the best model fit compared to plasma and CSF Aβ_42/40_ models (*P* < 0.05). Also, all ROC models were similar using the DeLong test, with AUC ranging from 0.66 to 0.75 for Aβ, from 0.74 to 0.81 for tau, and between 0.77 to 0.82 for their combinations (Figure 3).

Finally, tau biomarker cutoffs used in Figure 1, which are hypothesized as being optimal to identify AD pathology, gave good to excellent specificity (97% for plasma, 95% for CSF, and 100% for PET), but low sensitivity (34%, 45%, and 17%, respectively) at identifying CU who will develop MCI. Except for CSF, Aβ biomarker cutoffs had low specificity (66% for plasma, 94% for CSF, and 77% for PET), and low sensitivity (65%, 45%, and 55%, respectively). See Tables S7–S12 for the sensitivity, specificity, negative predictive value, and positive predictive value of all other possible cutoffs.

## 4 DISCUSSION

We assessed the performance of plasma p‐tau217, alone or combined with plasma Aβ_42/40_, at identifying CU individuals who will develop MCI in a 10‐year window (the mean follow‐up being 6 years after plasma measurement with follow‐ups ranging from 1 to 10 years). In the main analyses, p‐tau217 and Aβ_42/40_ were stratified, since stratification is important for clinical use. For instance, only individuals with Aβ pathology are enrolled in anti‐amyloid trials or can receive medication to slow down AD. For most diseases, clinical diagnosis is also based on a threshold delimitating what is considered in the normal range versus what is abnormal. Given that there are no universal thresholds for plasma markers, and because plasma values vary depending on analytic techniques, we also showed the results using continuous variables. Finally, the analyses were replicated using CSF and PET biomarkers, the latter being the gold standard to quantify amyloid and tau pathology in vivo.

For all biomarkers assessed, we found that A+T+ individuals had a higher risk of progression to MCI compared to the A−T− group. While only few individuals were classified as A−T+ across biomarkers, A−T+_plasma_ individuals were also at increased risk of progression to MCI compared to A−T−_plasma_ individuals. Supporting these results, the ROC analyses suggest that the accuracy of plasma p‐tau217 was similar to that found with CSF p‐tau217 or PET biomarkers and that the models were not improved when combining amyloid with the tau markers. Finally, all T+ participants developed MCI within 5 years when identifying T+ individuals based on both neocortical and medial temporal lobe regions (temporal meta‐ROI), but this T+ classification yielded > 50% fewer T+ participants than when exclusively using the entorhinal cortex or the p‐tau217 fluid biomarkers (Figure 1 and Figure S5). In line with these results, CU individuals with neocortical tau were found to be at increased risk of progression to dementia compared to CU individuals with medial temporal tau.^5^

Robust and accurate blood‐based markers for AD are needed for clinical evaluation, trial recruitment, and to identify individuals who could benefit from disease‐modifying therapies.^22^, ^23^, ^24^, ^25^ Plasma p‐tau217 has been found to differentiate CU from individuals with clinical AD;^26^ it can detect AD pathology in individuals with MCI;^27^ and it correlates with Aβ PET, tau PET, and cognitive decline in CU participants.^28^, ^29^, ^30^, ^31^, ^32^ Our prospective study shows that plasma p‐tau217 can also be used to predict the development of MCI in CU individuals years before cognitive onset. It also stresses the fact that CU individuals with tau pathology have a brain disease that will lead to cognitive impairments if not treated and that finding a preventive treatment for these individuals is therefore a priority.

Regardless of the biomarker, Aβ did not improve the predictive value of the tau biomarkers, even when using IP‐MS to quantify plasma, which is known to be a more accurate technique than immunoassay.^33^ This can mainly be explained by the fact that almost all T+ individuals were also A+.^34^ The finding is also consistent with previous studies that have demonstrated Aβ proteins plateau earlier in the AD spectrum, whereas p‐tau levels continue to increase in the prodromal stages of the disease making Aβ less useful for predicting cognitive changes.^35^ As for individuals with abnormal Aβ but normal tau biomarkers, their risk of progression to MCI was only increased when classified with temporal meta‐ROI tau PET compared to the A−T− group. This can be attributed to the fact that fluid biomarkers capture soluble and diffusible proteins, while PET images capture insoluble aggregates that are characteristic of later stages of the disease.^7^ Individuals classified as positive on an Aβ PET scan who are not yet positive on a tau PET scan probably have some level of tau that is not yet detected by a PET scan. This might be particularly true when T+ is defined based on neocortical regions.^5^ Supporting this hypothesis, 10% of the participants were tau positive on plasma p‐tau217 while negative on tau PET, and only 1% had the inverse profile. Most (79%) of these tau plasma positive and tau PET negative also developed MCI. Furthermore, 128 participants included in the current PET subsample were included in a previous publication. While only 9% of the A+T− participants had developed MCI after a follow‐up of 3.4 years in this previous publication, 2.4 years later, 42% of them (54/129) now have MCI.^4^ These results suggest that most A+T− PET participants will also develop cognitive impairments if followed for > 4 years. Such a conclusion cannot be made with fluid biomarkers. It is possible that plasma and CSF A+T− individuals are further away in the disease process, or that soluble Aβ is not sufficient to cause AD. Finally, the direct comparison between plasma and CSF Aβ_42/40_ suggests that plasma might become abnormal prior to CSF (Figure 2C), a finding that will need to be replicated.

Twenty‐seven percent of our participants developed MCI during a mean cognitive follow‐up of 7.7 years (SD = 1.93, range 1.39–10.49 years), which is a percentage similar to what has been found in a recent community‐based study.^36^ The unimpaired versus impaired cognitive classification was based on research data done blind to *APOE* ε4 status and MRI, CSF, and PET biomarker results. Not all MCI individuals are therefore on the path toward AD dementia. MCI can develop for multiple reasons and most A−T− individuals probably have non‐neurodegenerative conditions such as vascular or psychiatric conditions.^37^ Approximately 60% of individuals classified as MCI were later followed in a clinical setting unblind to biomarker status and one third of the A+T+ have now developed dementia while none of the A−T− followed in an affiliated clinical setting developed dementia and one third of the A−T− are now considered to only have subjective cognitive impairment (Figure S3). Finally, the selected thresholds were not sensitive at identifying all causes of MCI (Table S7–S12). The thresholds were selected to optimize the detection of AD pathology, not to detect MCI with no AD pathology. The sensitivity of all possible thresholds at identifying all causes of MCI can nevertheless be found in supporting information.

One main limitation is the low racial, ethnic (98% White), and sex imbalance (72% female) of our population, as the findings may not be generalizable to all populations. The low sample size in the A+T+ and the A−T+ groups should also be noted. The percentage of participants included in these groups is nevertheless similar to what was found in previous PET studies involving preclinical cohorts.^4^, ^5^ Given this limitation and the fact that 20% of our reference group developed MCI, the HRs between groups should be interpreted with caution. Finally, part of the study follow‐up occurred during the COVID‐19 pandemic, which may have contributed to the development or deterioration of cognitive impairment.^38^

In this longitudinal multimodal biomarker study of individuals with a first‐degree family history of AD dementia spanning > 10 years, 27% developed MCI based on a multidisciplinary classification consensus meeting blind to AD biomarkers and *APOE* genotype information. While not interchangeable, fluid and PET biomarkers of AD pathology are both extremely valuable for identifying individuals who will develop MCI, with almost all individuals with abnormal p‐tau217 values developing MCI within a 10‐year follow‐up. While these results suggest that p‐tau217 quantified in a specialized center can be used as a stand‐alone test to identify CU who will develop MCI, plasma and CSF biomarkers are known to be more prone to measurement errors, matrix effects, and batch‐to‐batch variability compared to PET.^39^, ^40^ Such limitations need to be considered when implementing plasma p‐tau217 in memory clinics. Given the possible distress caused by the disclosure of the biomarker results and the lack of preventive treatments, we also strongly advocate that people who do not yet have cognitive impairments should not be tested or diagnosed based on plasma (or PET) biomarkers in clinical settings, and restricting the use of plasma p‐tau217, CSF p‐tau217, and tau PET to research settings and specialized clinics where counseling is available.

## AUTHOR CONTRIBUTIONS

Yara Yakoub, Cherie Strikwerda‐Brown, Nicholas J. Ashton, Michael Schöll, Pedro Rosa‐Neto, Judes Poirier, John C. S. Breitner, Henrik Zetterberg, Kaj Blennow, and Sylvia Villeneuve contributed to the study concept and design. Yara Yakoub, Fernando Gonzalez‐Ortiz, Nicholas J. Ashton, Christine Dery, Frédéric St‐Onge, Valentin Ourry, Maiya Geddes, Simon Ducharme, Maxime Montembeault, and Jean‐Paul Soucy contributed to data acquisition and analysis. Yara Yakoub and Sylvia Villeneuve drafted the manuscript and figures.

## CONFLICT OF INTEREST STATEMENT

H.Z. has served on scientific advisory boards and/or as a consultant for Abbvie, Acumen, Alector, Alzinova, ALZPath, Amylyx, Annexon, Apellis, Artery Therapeutics, AZTherapies, Cognito Therapeutics, CogRx, Denali, Eisai, Merry Life, Nervgen, Novo Nordisk, Optoceutics, Passage Bio, Pinteon Therapeutics, Prothena, Red Abbey Labs, reMYND, Roche, Samumed, Siemens Healthineers, Triplet Therapeutics, and Wave; has given lectures in symposia sponsored by Alzecure, Biogen, Cellectricon, Fujirebio, Lilly, Novo Nordisk, and Roche; and is a co‐founder of Brain Biomarker Solutions in Gothenburg AB (BBS), which is a part of the GU Ventures Incubator Program (outside submitted work). M.S. has served on advisory boards for Roche, Novo Nordisk, and Servier; received speaker honoraria from Bioarctic, Eisai, Genentech, Novo Nordisk, and Roche; and receives research support (to the institution) from Alzpath, Bioarctic, Novo Nordisk, and Roche (outside scope of submitted work). He is a co‐founder of Centile Bioscience Ltd. No other disclosures were reported. Author disclosures are available in the supporting information.

## CONSENT STATEMENT

Written informed consent was obtained for all PREVENT‐AD participants. All research procedures were approved by the institutional review board at McGill University.

## CODE AVAILABILITY

The code used for the statistical analyses is available from the first author upon request. The analyses and figures were built using R programming language (v.4.2.2) and R studio “Elsbeth Geranium” Release (7d165dcfc1b6d300eb247738db2c7076234f6ef0, 2022‐12‐03) for macOS (packages used for the main analyses: survival v3.4‐0; survminer v0.4.9; lme4 v1.1‐31; lmerTest v3.1‐3; ggplot v2.3.5.0; pROC v1.18.4).

## Supporting information



Supporting Information

Supporting Information

## Data Availability

Data used in the preparation of this manuscript were obtained from the Pre‐symptomatic Evaluation of Experimental or Novel Treatments for Alzheimer's Disease (PREVENT‐AD). Some of the data are publicly available (https://openpreventad.loris.ca and https://registeredpreventad.loris.ca), and the remaining data can be shared upon approval by the scientific committee at the Centre for Studies on Prevention of Alzheimer's Disease (StoP‐AD) at the Douglas Mental Health University Institute.

## 1

A list of all PREVENT−AD collaborators can be found at the following website https://preventad.loris.ca/acknowledgements/acknowledgements.php?date=2024‐12‐02&authors.
