# Supplementary material for: Plasma p‐tau217 identifies cognitively normal older adults who will develop cognitive impairment in a 10‐year window
Source: Alzheimers Dement. 2025 Feb 26;21(2):e14537. doi: 10.1002/alz.14537 (PMC11863240; doi:10.1002/alz.14537)
Supplement: Supplementary file 1 — Supporting Information [file ALZ-21-e14537-s002.docx]

**Supplemental Online Content**

**Plasma p-tau217 identifies cognitively normal older adults who will develop cognitive impairment in a 10-year window**

Yara Yakoub, BSc^1^; Fernando Gonzalez-Ortiz, MD^2^; Nicholas J. Ashton, PhD^2,3,4,5^; Christine Déry, MSc^1^; Cherie Strikwerda-Brown, PhD^1,6^; Frédéric St-Onge, PhD^1^; Valentin Ourry, PhD^1^; Michael Schöll, PhD ^2,3,7^; Maiya R. Geddes, MD ^8,9,10^; Simon Ducharme, MD, MSc ^1,8^; Maxime Montembeault, PhD^1,11^; Pedro Rosa-Neto, MD^8,10^; Jean-Paul Soucy, MD, MSc^8^; John C.S. Breitner, MD, MPH^1,11^; Henrik Zetterberg, MD, PhD^2,7,12,13,14,15^; Kaj Blennow, MD, PhD^2,12^; Judes Poirier, PhD ^1,11^; Sylvia Villeneuve, PhD ^1,8,11^; PREVENT-AD Research Group.

^1^ Douglas Mental Health University Institute, Centre for Studies on the Prevention of

Alzheimer's Disease (StoP-AD), Montreal, Quebec, Canada. 6875 Blvd. LaSalle, Verdun, H4H 1R3, Quebec, Canada

^2^ Department of Psychiatry and Neurochemistry, Institute of Neuroscience and Physiology, The

Sahlgrenska Academy, University of Gothenburg, Gothenburg, Sweden. Blå Stråket 15, vån 3 SE-413 45 Göteborg Sweden

^3^ Centre for Age-Related Medicine, Stavanger University Hospital, Stavanger, Norway. Armauer Hansens vei 30, 4011 Stavanger, Norway

^4^ King’s College London, Institute of Psychiatry, Psychology & Neuroscience, Maurice Wohl

Clinical Neuroscience Institute, London, UK. 16 De Crespigny Park, London SE5 8AF, United Kingdom

^5^ Banner Alzheimer’s Institute, Phoenix, AZ, USA

^6^ School of Psychological Sciences, The University of Western Australia, Perth, Australia. 35 Stirling Highway Perth, Crawley WA 6009, Australia

^7^ Department of Neurodegenerative Disease, UCL Queen Square Institute of Neurology,

University College London, London, UK. Queen Square, London WC1N 3BG, United Kingdom

^8^ Montreal Neurological Institute, McGill University, Montreal, Quebec, Canada. 3801 Rue University, Montréal, QC H3A 2B4, Quebec, Canada

^9^ McGovern Institute for Brain Research, Massachusetts Institute of Technology, Cambridge, MA, USA. 77 Massachusetts Ave, Cambridge, MA 02139, United States

^10^ McGill University Research Centre for Studies in Aging, McGill University, Montreal, Quebec, Canada. 6825 Blvd. LaSalle, Verdun, H4H 1R3, Quebec, Canada

^11^ Department of Psychiatry, McGill University, Montreal, Quebec, Canada. 1033 Pine Avenue West Montreal, H3A 1A1, Quebec, Canada

^12^ Clinical Neurochemistry Laboratory, Sahlgrenska University Hospital, Mölndal, Sweden. Wallinsgatan 6, 431 39 Mölndal, Sweden

^13^ UK Dementia Research Institute at UCL, London, UK. Gower St, London WC1E 6BT, United Kingdom

^14^ Hong Kong Center for Neurodegenerative Diseases, Clear Water Bay, Hong Kong. Units 1501-1502, 1512-1518, 15/F Building 17W, 17 Science Park W Ave, Science Park, Hong Kong

^15^ UW Department of Medicine, School of Medicine and Public Health, Madison, WI, USA. 750 Highland Avenue Madison, WI 53705

**Correspondence**

Sylvia Villeneuve, Douglas Mental Health University Institute, Centre for Studies on the Prevention of Alzheimer’s Disease (StoP-AD), Perry Pavilion Room E3417.1, 6875 Boulevard LaSalle, Montreal, QC H4H1R3, Canada. Email: Sylvia.villeneuve@mcgill.ca

**Key words:** MCI, PET, CSF, Plasma, Amyloid, Tau

**Total word count of the main text in Supplement**: 2537

**The supplementary includes** 14 references, 12 Tables and 6 Figures

**Table of contents**

**Methods S1**

**Results S1**

**Supplementary References**

**Table S1. Demographic Characteristics of participants across AT plasma full sample**

**Table S2. Demographic Characteristics of participants across AT CSF full sample**

**Table S3. Demographic Characteristics of participants across AT PET full sample**

**Table S4. Demographic Characteristics of participants across AT plasma subsample**

**Table S5. Demographic Characteristics of participants across AT CSF subsample**

**Table S6. Demographic Characteristics of participants across AT PET subsample**

**Table S7. Diagnostic accuracy of plasma p-tau217 in predicting MCI**

**Table S8. Diagnostic accuracy of plasma Aβ_42/40_ in predicting MCI**

**Table S9. Diagnostic accuracy of CSF p-tau217 in predicting MCI**

**Table S10. Diagnostic accuracy of CSF Aβ_42/40_ in predicting MCI**

**Table S11. Diagnostic accuracy of meta-ROI tau-PET in predicting MCI**

**Table S12. Diagnostic accuracy of Aβ -PET in predicting MCI**

**Figure S1. Flowchart of participants included in the study.**

**Figure S2. Distribution of plasma, CSF, PET, and cognitive measurements.**

**Figure S3. Severity of the cognitive impairment based on clinical setting evaluations unblind to biomarker status.**

**Figure S4. Clinical progression to MCI in the subsample of 93 individuals with all AT biomarkers.**

**Figure S5. Clinical progression to MCI across PET AT biomarker groups in the full sample and subsample using entorhinal cortex to define tau positivity.**

**Figure S6. Percentage of cognitively unimpaired vs MCI individuals between plasma, CSF and PET Aβ and tau biomarkers.**

**Methods S1**

# **PREVENT-AD cohort**

The PREVENT-AD cohort is an ongoing longitudinal observational study of older adults with self-reported parental or multiple-sibling history of sporadic AD. ^1^ REVENT-AD participants were enrolled between 2011 and 2017, were 60+ years old, or 55-59 if within 15 years of their youngest-affected relative’s age of onset and CU at enrollment.^1^ Information on *APOE ε4* status, age, sex, years of education were collected at entry into the program. Normal cognition at enrollment was based on a brief cognitive screening at study entry using the Clinical Dementia Rating (CDR) and Montreal Cognitive Assessment (MoCA). In a few cases of ambiguous CDR (0.5) or MoCA (≤26), participants were evaluated by a certified neuropsychologist with an extensive neuropsychological battery assessment. Participants were then followed annually using the Repeatable Battery for the Assessment of Neuropsychological Status (RBANS) and other cognitive tasks.^1^ Individuals at baseline who performed below the norms for the RBANS were also evaluated by a certified neuropsychologist to confirm normal cognition. Blood draws were collected at baseline and up to four years of follow up (2011-2017). Information on peripheral blood Aβ40 and Aβ42 concentrations, measured using IP-MS, was available for a subset of PREVENT-AD participants with either CSF or PET measurements obtained up to 2017. CSF collection via lumbar puncture, when available, followed a similar timeline to plasma, while PET was introduced in 2017. Overall, we included 215 participants with available plasma, 159 with available CSF and 155 with available PET measurements (**Figure S1**). In our study, 143 participants had plasma and PET available with mean time difference of 1.33 years (SD = 1.58, range 0.38 – 7.73 years). Plasma measurements were performed earlier than PET except for 13 participants who had plasma measurements following the PET scans (6- 137 days). Similarly, 93 participants had CSF and PET available with mean time difference of 1.44 years (SD = 1.73, range 0.57 – 7.73 years) apart from ten participants who received PET prior to CSF measurements with 6-209 days. One hundred fifty-eight participants had CSF and plasma available with mean time difference of 0.21 years (SD = 0.70, range 3.98 -4.08 years) with eight participants having CSF collected prior to plasma (1 – 1453 days). For our study, we utilized plasma and CSF data collected from 2012-2017, PET data spanning from 2017-2023, cognitive data from 2012 up to 2023, and the related available MCI classification (**Figure S2**).

**Plasma measures**

In the plasma sample, Aβ40 and Aβ42 concentrations were measured using ultrasensitive immunoprecipitation coupled with mass spectrometry (IP-MS) technique using a KingFisher Flex Purification System (Thermo Fisher Scientific). ^2^ Inter-assay coefficients of variation were <5%.^2^ In brief, Aβ peptides were extracted from 0.25 ml samples using immunoprecipitation with anti-Aβ antibodies 4G8 bound to IgG magnetic beads (Thermo Fisher).^2^ Immunoprecipitation was automated with the KingFisher Flex system. Eluates in 0.5% formic acid were vacuum-centrifuged, stored at −80°C, reconstituted in 20% acetonitrile with 4% ammonia, and analyzed by LC-MS/MS (Dionex Ultimate LC and Thermo Q Exactive).^2^ Quantification was then performed with liquid chromatography/high-resolution parallel reaction monitoring (PRM) mode.^2^

Plasma p-tau217 concentrations were measured using an in-house Simoa platform developed at the Clinical Neurochemistry Laboratory, University of Gothenburg.^3^ Prior to analysis, the plasma samples were thawed, vortexed, and centrifuged (4000g for 10 min at room temperature). They were then examined using an HD-X analyzer, with identical reagent batches used throughout the study. To ensure quality control, two quality control (QC) plasma samples were included at the beginning and end of each run, resulting in an overall intraplate variation and intermediate precision of < 10% for the biomarker measurement. All plasma samples were measured by scientists blinded to participants’ clinical information.

**Cerebrospinal Fluid (CSF) measures**

A total of 159 PREVENT-AD participants underwent a lumbar puncture (LP) procedure after fasting overnight. A Sprotte 24-gauge atraumatic needle was used. Approximately of 20–30 mL were centrifuged at room temperature for 10 min at ~ 2000g, then aliquoted into 0.5 mL polypropylene cryotubes, and quick-frozen at −80 °C for long-term storage.^1, 4^ CSF Aβ1-42, Aβ1-40 were measured using Lumipulse G- automated immunoassay. ^5^ CSF P-tau217 was measured using an in-house Simoa platform developed at the Clinical Neurochemistry Laboratory, University of Gothenburg.^3^ All CSF samples were measured by scientists blinded to participants’ clinical information. Intraplate variation was determined with internal quality controls at the beginning and end of the plate (CV 5.8%). Intermediate precision was determined with quality controls at the beginning of each plate (CV 14.4%).

**PET acquisition and preprocessing**

The PET scans were performed at the McConnell Brain Imaging Centre of the Montreal Neurological Institute, Canada, using a brain-dedicated PET Siemens/CTI high-resolution research tomograph. Aβ-PET images using ^18^F-NAV4694 as tracer were captured 40-70 minutes after injecting a targeted dose of 220 MBq (6 mCi). Tau-PET images using ^18^F-flortaucipir as tracer were obtained 80-100 minutes after injection with a targeted dose of 370 MBq (10 mCi). We acquired 6 frames of 5 minutes for NAV and 4 frames of 5 minutes for FTP. A transmission attenuation correction scan was also performed. The images were reconstructed using a three-dimensional (3D) ordinary Poisson ordered subset expectation maximum ([OP-OSEM], with 10 iterations and 16 subsets).^6, 7^ Decay and motion corrections were applied to the images, and scatter correction was done using a 3D scatter estimation method.^7^ The MRI scan closest in time to the PET scan for each participant was selected for PET image preprocessing. The T1-weighted MRI images were preprocessed and divided into 34 bilateral regions of interest (ROI) based on the Desikan-Killiany atlas using FreeSurfer v.5.3.^8^ The PET images were realigned, temporally averaged, and co-registered to the closest T1-weighted image. Then, they were masked to remove CSF signal and smoothed using a 6-mm^3^ Gaussian kernel. Standardized Uptake Value Ratios (SUVRs) were calculated as the ratio of tracer uptake in the regions of interest compared to the uptake in the gray matter of the cerebellum for Aβ-PET scans or to the inferior cerebellar gray matter for tau-PET scans. ^9, 10^ Global amyloid index was calculated by averaging bilateral SUVR of the precuneus, cingulate, medial and lateral frontal, parietal and temporal cortical regions.^9, 11^ For tau, the temporal meta-ROI was calculated as the average SUVR from the bilateral entorhinal cortex, fusiform, parahippocampal, inferior temporal and middle temporal gyri and amygdala.^12^ An in-house pipeline (https://github.com/villeneuvelab/vlpp) was used for the preprocessing of all PET scans. The PET data underwent a new reconstruction when compared to the Strikwerda-Brown et al. study published in 2022.^13^ The PET sample overlaps with the former study, with addition of twenty-seven participants, all of whom had scans taken between 2018 and 2021.

**Clinical status**

After their MCI classification most participants (38/62) were referred and followed by a physician in a clinical setting. The evaluation in the clinical setting was always performed after the research classification and **Figure S3** represents the most updated clinical status as of November 2024. Available information is based on the clinical chart for participants who have been seen and/or followed in an affiliate memory clinic (n=36), information provided by an external clinic (n=1) and information provided by a spouse (n=1). All A+T+ received a clinical diagnosis of AD based on their biomarker status. None of the A-T- received a diagnosis of AD and the suspected cause of cognitive impairment were vascular, dysthymic or uncertain. The etiology of the A+T- and A-T+ was less uncertain with one participant who was hypothesized to have frontotemporal dementia. Among the A-T- classified as having subjective cognitive impairment one was suspected to have a neurodevelopmental condition.

**Results S1**

**Rate of progression from CU to MCI across AT groups when defined using fluid versus neuroimaging biomarkers.**

We replicated the same analyses using the subsample of 93 participants with plasma, CSF, and PET measurements. In the plasma subsample, the results showed that 9% (8/93) were classified as A+T+, 35% (33/93) as A+T-, 3% (3/93) as A-T+ and 53% (49/93) as A-T-. Using Aβ_42/40_ and p-tau217 CSF biomarkers, 15% (14/93) were classified as A+T+, 3% (3/93) as A+T-, 2% (2/93) as A-T+ and 80% (74/93) as A-T-. Finally, using PET biomarkers, 3% (3/93) were classified as A+T+, 30% (28/93) as A+T-, 1% (1/93) as A-T+ and 66% (61/93) as A-T-. These numbers were not statistically different to the proportion found in the full sample (Fisher’s exact p > 0.05) which is similar to what has been found in other PET cohorts.^13, 14^

Eighty-eight percent (7/8) of the A+T+_plasma_ group developed MCI, compared with 36% (12/33) in the A+T- _plasma_ group, 100% (3/3) in the A-T+ _plasma_ group and 14% (7/49) in the A-T- _plasma_ group (**Figure S4A**). The proportion of CU developing MCI was higher in the A+T+ group when compared with A-T-_plasma_ and A+T-_plasma_ groups (Fisher’s exact p < 0.001, p = 0.01). We also found that A+T-_plasma_ and A-T+_plasma_ groups were significantly different from the reference group (Fisher’s exact p = 0.03, p = 0.005). When the groups were classified using CSF, 86% (12/14) of the A+T+ _CSF_, 33% (1/3) of the A+T- _CSF_ group, 50% (1/2) of the A-T+ and 20% (15/74) of the A-T- _CSF_ group developed MCI (**Figure S4B**). An increased CU to MCI progression rate was found in the A+T+_CSF_ group when compared A-T-_CSF_ (Fisher’s exact p < 0.001), but no differences were found between A+T-_CSF_ and the reference group. In the PET groups, 100% (3/3) of A+T+ _PET_ biomarker group, 46% (13/28) of the A+T- _PET_ group, 100% (1/1) of the A-T+ and 20% (12/61) of the A-T- _PET_ group progressed to MCI (**Figure S4C**). The A+T+ _PET_ and A+T- _PET_ groups were associated with increased progression to MCI when compared with A-T- _PET_ (Fisher’s exact p = 0.01, p = 0.01 respectively).

Cox proportional hazard models showed a higher risk of progression from CU to MCI among the A+T+_plasma_ and A-T+ _plasma_ (hazard ratios (HR) = 6.61, p = 0.001, 95%CI = 2.06 – 21.17; HR = 6.25; p = 0.01, 95%CI = 1.53- 25.54; model concordance value (model fit) = 0.69**;** SE = 0.07; **Figure S4D & G**) when compared to A-T- _plasma_ (reference) group. No differences in the risk of progression were found between A+T- _plasma_ and the reference group (HR = 1.72, p = 0.30, 95%CI = 0.62 – 4.82) In the CSF sample, we found an increased in the risk among A+T+_CSF_ (HR = 3.63, p = 0.005, 95%CI = 1.49 – 8.84; model concordance value = 0.71; SE = 0.07; **Figure S4E & H)** compared to A-T-_CSF_ group while no differences in the risk of progression found between A+T-_CSF_ and A-T-_CSF_ (HR = 2.03, p = 0.51, 95%CI = 0.24 – 16.90). Finally, A+T+_PET_ participants exhibited a higher risk of MCI progression compared to A-T-_PET_ and A+T-_PET_  (HR = 9.16, p = 0.001*,* 95% CI = 2.33 – 36.01; HR = 2.60, p = 0.034, 95%CI = 1.07 – 6.31; model concordance value = 0.71; SE = 0.06; **Figure S4F & I**). Groups with fewer than three participants were excluded from the analyses; this includes the A-T+ groups in both the CSF and PET samples.

We also investigated the longitudinal cognitive performance of participants within the AT biomarker groups while taking advantage of all cognitive time points, including those before the biomarker classifications, over a mean of 8.06 years (SD 1.68, range 2.99 – 10.47). The A+T+_plasma_ and A-T+_plasma_ groups demonstrated a steeper cognitive decline compared to A-T-_plasma_ (reference) group (β = -0.79, p = 0.04, SE = 0.39, %95CI = -1.56 – -0.02; β = -1.63, p = 0.004, SE =0.55, 95%CI = -2.72 – -0.54; R^2^ = 0.08; **Figure S4J)** while no differences was observed between the A-T-_plasma_ and A+T-_plasma_ group (β = 0.06, p = 0.74, SE = 0.19, %95CI = -0.32 – 0.46). The A+T+_CSF_ group showed faster decline over time compared to A-T-_CSF_ (β = -1.23, p < 0.001, SE = 0.27, 95%CI = -1.77 – -0.70; R^2^ = 0.13, **Figure S4K**), but no difference was found between the reference group and A+T-_CSF_ group (β = 0.48, p = 0.38, SE = 0.54, 95% CI = -0.59 – 1.56). The A+T+_PET_ group demonstrated a steeper cognitive decline compared to A-T-_PET_ (β = -1.85, p = 0.001, SE = 0.55, 95%CI = -2.94 – -0.76; **Figure S4L**). A+T^_^_PET_ group demonstrated no differences when compared to A-T-_PET_ group (β = -0.34, p = 0.09, SE = 0.20, 95%CI = -0.73 – 0.06).

**Rate of progression from CU to MCI across AT PET groups, using the entorhinal cortex to define tau positivity**

Eighty-nine percent (16/18) of the A+T+_EC_ group developed MCI, compared with 34% (12/35) in the A+T-_EC_ group, 50% (1/2) in the A-T_EC_+ group, and 20% (20/100) in the A-T-_EC_ group (**Figure S5A**). In the subsample of 93 participants with all biomarker measurements, 91% (10/11) of the A+T+_EC_, 30% (6/20) in the A+T-_EC_ group, 50% (1/2) in the A-T+_EC_ group, and 20% (12/60) in the A-T-_EC_ group (**Figure S5B**) progressed to MCI. In both samples, the A+T+_EC_ group was associated with increased progression to MCI compared with the A-T-_EC_ and A+T- _EC_ groups (Fisher’s exact p values < .05).

In the sample of 155 participants, cox proportional hazard models showed a higher risk of progression of A+T+_EC_ compared to A-T-_EC_ group while no differences were observed between the A+T-_EC_ group and the reference group (HR = 6.94, p < 0.001*,* 95%CI = 3.11 – 15.45; HR = 2.13, p = 0.05, 95%CI = 1.01 - 4.50; model concordance value = 0.73; SE = 0.04; **Figure S5C&E**). The findings in the subsample of 93 participants were similar, with higher risk of progression was observed between A+T+_EC_ group and the A-T-_EC_ group while no differences were found between the A+T-_EC_ group and the reference group (HR = 6.79, p < 0.001*,* 95%CI = 2.33 – 19.81; HR = 1.65, p = 0.36, 95%CI = 0.57 - 4.83; model concordance value = 0.72; SE = 0.06; **Figure S5D&F**).

Consistent with our main findings using the temporal meta-ROI to define tau-PET positivity, A+T+_EC_ participants exhibited greater longitudinal cognitive decline compared with the reference group (Full sample: β = -1.39, p < 0.001, SE = 0.25, 95%CI = -1.88 - -0.90; Subsample: β = -1.32, p < 0.001, SE = 0.29, 95%CI = -1.91 - -0.75; **Figure S5G-H**). However, there were no differences in cognitive trajectories between A+T-_EC_ group compared to A-T-_EC_ (Full sample: β = -0.03, p = 0.84, SE = 0.17, 95%CI = -0.38 - 0.30; Subsample: β = -0.06, p = 0.77 , SE = 0.22, 95%CI = -0.50 - 0.37; **Figure S5G-H**)

**Concordance between the different biomarkers.**

When these biomarkers were stratified by +/- status (**Figure S6**), Aβ plasma and PET biomarkers were concordant for 69% of the participants (22% Plasma+/PET+; 47% Plasma-/PET-) with a similar number of discrepancies between the Plasma-/PET+ (13%) and the Plasma+/PET- (18%). The concordance between plasma p-tau217 and tau PET was 89% (5% Plasma+/PET+; 84% Plasma-/PET-), with 1% Plasma-/PET+ and 10% Plasma+/PET-. The concordance between plasma and CSF Aβ_42/40_ was 70% (12% Plasma+/CSF+; 58% Plasma-/CSF-), with 3% Plasma-/CSF+ and 27% Plasma+/CSF-. The concordance between plasma and CSF p-tau217 was 92% (8% Plasma+/CSF+; 84% Plasma-/CSF-), with 6% Plasma-/ CSF+ and 2% Plasma+/ CSF-. The concordance between Aβ CSF and PET was 81% (16% CSF+/PET+; 65% CSF-/PET-), with 17% CSF-/PET+ and 2% CSF+/PET-. The concordance between CSF p-tau217 and tau-PET was 87% (4% CSF+/ PET+; 83% CSF-/ PET-), with 0% CSF-/PET+ and 13% CSF+/PET-.

**Supplementary References**

1. Tremblay-Mercier J, Madjar C, Das S, et al. Open science datasets from PREVENT-AD, a longitudinal cohort of pre-symptomatic Alzheimer's disease. *NeuroImage Clinical*. 2021;31:102733. doi:10.1016/j.nicl.2021.102733

2. Meyer P-F, Ashton NJ, Karikari TK, et al. Plasma p-tau231, p-tau181, PET Biomarkers, and Cognitive Change in Older Adults. *Annals of neurology*. 2022;91(4):548-560. doi:10.1002/ana.26308

3. Gonzalez-Ortiz F, Ferreira PCL, González-Escalante A, et al. A novel ultrasensitive assay for plasma p-tau217: Performance in individuals with subjective cognitive decline and early Alzheimer's disease. *Alzheimers Dement*. Feb 2024;20(2):1239-1249. doi:10.1002/alz.13525

4. Meyer P-F, Savard M, Poirier J, et al. Bi-directional Association of Cerebrospinal Fluid Immune Markers with Stage of&nbsp;Alzheimer’s Disease Pathogenesis. *Journal of Alzheimer's Disease*. 2018;63:577-590. doi:10.3233/JAD-170887

5. Gobom J, Parnetti L, Rosa-Neto P, et al. Validation of the LUMIPULSE automated immunoassay for the measurement of core AD biomarkers in cerebrospinal fluid. *Clin Chem Lab Med*. Jan 27 2022;60(2):207-219. doi:10.1515/cclm-2021-0651

6. Varrone A, Sjöholm N, Eriksson L, Gulyás B, Halldin C, Farde L. Advancement in PET quantification using 3D-OP-OSEM point spread function reconstruction with the HRRT. *European Journal of Nuclear Medicine and Molecular Imaging*. 2009/10/01 2009;36(10):1639-1650. doi:10.1007/s00259-009-1156-3

7. Sibomana M, Keller S, Stute S, Comtat C. *Benefits of 3D scatter correction for the HRRT - a large axial FOV PET scanner*. 2012:2954-2957.

8. Desikan RS, Ségonne F, Fischl B, et al. An automated labeling system for subdividing the human cerebral cortex on MRI scans into gyral based regions of interest. *NeuroImage*. 2006;31(3):968-80.

9. Villeneuve S, Rabinovici GD, Cohn-Sheehy BI, et al. Existing Pittsburgh Compound-B positron emission tomography thresholds are too high: statistical and pathological evaluation. 2015;138(7):2020-2033. doi:10.1093/brain/awv112

10. Baker SL, Maass A, Jagust WJ, Lawrence Berkeley National Lab BCA. Considerations and code for partial volume correcting [ 18 F]-AV-1451 tau PET data. *Data in Brief*. 2017;15(C)doi:10.1016/j.dib.2017.10.024

11. Qiu T, Liu Z-Q, Rheault F, et al. Structural white matter properties and cognitive resilience to tau pathology. *Alzheimer's & Dementia*. 2024/05/01 2024;20(5):3364-3377. doi:<https://doi.org/10.1002/alz.13776>

12. Jack CR, Jr., Wiste HJ, Weigand SD, et al. Defining imaging biomarker cut points for brain aging and Alzheimer's disease. *Alzheimers Dement*. Mar 2017;13(3):205-216. doi:10.1016/j.jalz.2016.08.005

13. Strikwerda-Brown C, Hobbs DA, Gonneaud J, et al. Association of Elevated Amyloid and Tau Positron Emission Tomography Signal With Near-Term Development of Alzheimer Disease Symptoms in Older Adults Without Cognitive Impairment. *JAMA Neurology*. 2022;doi:10.1001/jamaneurol.2022.2379

14. Ossenkoppele R, Pichet Binette A, Groot C, et al. Amyloid and tau PET-positive cognitively unimpaired individuals are at high risk for future cognitive decline. *Nature Medicine*. 2022;28(11):2381-2387. doi:10.1038/s41591-022-02049-x

**Tables**

**Table S1. Demographic Characteristics of participants across AT plasma full sample.**

|  | **A+T+**  **(n = 21)** | **A+T-**  **(n = 60)** | **A-T+**  **(n = 7)** | **A-T-**  **(n = 127)** | **Group differences** |
| --- | --- | --- | --- | --- | --- |
| **Age at baseline, years** | 65.99 (4.90) | 63.99 (5.44) | 64.94 (4.72) | 62.59 (4.41) | p = 0.03^c^ |
| **Age at plasma visit, years** | 68.07 (4.63) | 65.46 (5.83) | 66.53 (5.21) | 64.46 (4.97) | p = 0.01^c^ |
| **Sex, F, n (%)** | 12 (57) | 41 (68) | 6 (86) | 98 (77) | p = 0.17 |
| **Education, years** | 15.10 (3.40) | 14.52 (2.92) | 16 (3.31) | 15.65 (3.31) | p = 0.19 ^c^ |
| ***APOE* ε4 carriers, n (%)** | 16 (76) | 25 (42) | 5 (71) | 42 (33) | p <0.001 ^a,c^ |
| **Aβ_42/40_** | 0.08 (0.01) | 0.08 (0.01) | 0.10 (0.01) | 0.10 (0.01) | p <0.001 ^b,c,d,f^ |
| **pTau217**  **(pg/ml)** | 5.19 (1.05) | 2.19 (0.73) | 6.50 (4.42) | 2.20 (0.74) | p <0.001 ^a,c,d,f^ |
| **MoCA score**  **/30** | 27.67 (1.68) | 28.03 (1.59) | 28.86 (0.90) | 28.17 (1.56) | p = 0.34 |
| **RBANS global score** | 99.41 (12.16) | 101.67 (9.93) | 95.86 (6.59) | 102.19 (9.49) | p = 0.24 |
| **MMSE score**  **/30** | 28.56 (1.31) | 28.83 (1.24) | 28.33 (1.37) | 28.87 (1.23) | p = 0.56 |

**Table S2. Demographic Characteristics of participants across AT CSF full sample.**

|  | **A+T+**  **(n = 18)** | **A+T-**  **(n = 5)** | **A-T+**  **(n = 3)** | **A-T-**  **(n = 133)** | **Group differences** |
| --- | --- | --- | --- | --- | --- |
| **Age at baseline, years** | 63.83 (4.80) | 65.20 (4.98) | 64.54 (3.98) | 62.67 (4.82) | p = 0.33 |
| **Age at CSF visit, years** | 66.30 (5.09) | 67.87(4.97) | 67.92(4.57) | 64.35(5.25) | p = 0.07 |
| **Sex, F, n (%)** | 9 (50) | 3 (60) | 1 (33) | 100 (75) | p = 0.04^c^ |
| **Education, years** | 13.50 (2.94) | 14.40 (3.36) | 17.33 (2.52) | 15.35 (3.16) | p = 0.03 |
| ***APOE* ε4 carriers, n (%)** | 16 (89) | 1 (20) | 1 (33) | 44 (44) | p < 0.001 ^a,c^ |
| **Aβ_42/40_** | 0.05 (0.01) | 0.06 (0.01) | 0.09 (0.01) | 0.10 (0.01) | p < 0.001 ^a,c^ |
| **pTau217**  **(pg/ml)** | 663.89 (125.89) | 278.88 (48.95) | 426.13 (14.92) | 190.96 (79.82) | p < 0.001 ^c,f^ |
| **MoCA score**  **/30** | 28.17 (1.29) | 28.41 (1.14) | 29.67 (0.58) | 27.98 (1.63) | p = 0.23 |
| **RBANS global score** | 97.94 (10.71) | 105.80 (5.17) | 98.00 (4.00) | 101.42 (9.83) | p = 0.19 |
| **MMSE score**  **/30** | 28.19 (1.80) | 29.00 (1.15) | 29.67 (0.58) | 28.86 (1.19) | p = 0.33 |

**Table S3. Demographic Characteristics of participants across AT PET full sample.**

|  | **A+T+**  **(n = 8)** | **A+T-**  **(n = 45)** | **A-T+**  **(n = 1)** | **A-T-**  **(n = 101)** | **Group differences** |
| --- | --- | --- | --- | --- | --- |
| **Age at baseline, years** | 69.02 (5.24) | 63.66 (4.83) | 62.90 | 63.30 (4.27) | p = 0.02 ^a,c^ |
| **Age at PET, years** | 72.58 (4.22) | 67.41 (5.24) | 64.42 | 67.38 (4.81) | p = 0.02 ^a,c^ |
| **Sex, F, n (%)** | 7 (88) | 33 (73) | 1 (100) | 70 (70) | p = 0.58 |
| **Education, years** | 15.25 (2.60) | 14.62 (2.79) | 11 | 15.69 (3.43) | p = 0.13 |
| ***APOE* ε4 carriers, n (%)** | 6 (75) | 27 (60) | 1(100) | 28 (28) | p < 0.001^c,e^ |
| **Global Aβ SUVR** | 1.98 (0.40) | 1.54 (0.30) | 1.25 | 1.16 (0.06) | p < 0.001^c,e^ |
| **Temporal meta-ROI SUVR** | 1.48 (0.13) | 1.16 (0.06) | 1.55 | 1.12 (0.07) | p < 0.001^a,c,e^ |
| **MoCA score**  **/30** | 28.62 (1.41) | 28.20 (1.66) | 28 | 28.08 (1.47) | p = 0.54 |
| **RBANS global score** | 94.45 (14.65) | 103.33 (9.78) | 86.00 | 102.99 (9.60) | p = 0.34 |
| **MMSE score**  **/30** | 27.75 (1.39) | 28.98 (1.27) | 29.00 | 28.85 (1.20) | p = 0.04 ^a,c^ |

**Table (S1 – 3)** represents the characteristics of plasma, CSF, and PET full sample and their corresponding AT groups. Data presented as mean (standard deviation), except for categorical variables where the count and percentage are presented. Fisher or Kruskal-Wallis test was performed between the groups and p values are reported on the right column. If a significant group difference (p<0.05) was found post-hoc p values are reported (^a^ difference between A+T+ and A+T-; ^b^ difference between A+T+ and A-T+; ^c^ difference between A+T+ and A-T-; ^d^ difference between A+T- and A-T+; ^e^ A+T- and A-T-; ^f^ A-T+ and A-T-). Age at baseline and at biomarker measurement are presented; RBANS values are shown at baseline. MoCA scores were collected at entry into the program. MoCA score was missing for one participant in the plasma and CSF

A-T- group. Abbreviations: MoCA = Montreal Cognitive Assessment; F = female; APOE = apolipoprotein E; SUVR = Standardized Uptake Value Ratio; RBANS = Repeatable Battery for the Assessment of Neuropsychological Status.

**Table S4. Demographic Characteristics of participants across AT plasma subsample.**

|  | **A+T+**  **(n = 8)** | **A+T-**  **(n = 33)** | **A-T+**  **(n = 3)** | **A-T-**  **(n = 49)** | **Group differences** |
| --- | --- | --- | --- | --- | --- |
| **Age at baseline, years** | 63.23 (4.76) | 63.82 (5.11) | 64.94 (2.02) | 62.59 (4.14) | p = 0.92 |
| **Age at plasma visit, years** | 65.38 (4.63) | 66.19 (5.18) | 63.70 (0.48) | 64.91 (4.66) | p = 0.79 |
| **Sex, F, n (%)** | 1(13) | 25 (76) | 3 (100) | 36 (73) | p = 0.003 ^a,b,e^ |
| **Education, years** | 13.38 (2.77) | 13.73 (2.55) | 14.67 (4.04) | 15.86 (2.77) | p = 0.006 ^e^ |
| ***APOE* ε4 carriers, n (%)** | 7 (88) | 14 (42) | 2 (67) | 16 (33) | p = 0.02 ^a,c^ |
| **Aβ_42/40_** | 0.08 (0.01) | 0.08 (0.01) | 0.10 (0.02) | 0.10 (0.01) | p < 0.001 ^b,c,d,e^ |
| **pTau217**  **(pg/ml)** | 5.39 (0.98) | 2.15 (0.70) | 5.45 (0.33) | 2.27 (0.67) | p < 0.001 ^a,c,d,f^ |
| **MoCA score**  **/30** | 27.50 (1.41) | 28.06 (1.66) | 29.00 (1.00) | 28.10 (1.34) | p = 0.45 |
| **RBANS**  **global score** | 100.00 (12.27) | 102.42 (10.34) | 95.33 (8.08) | 102.14 (9.11) | p = 0.53 |
| **MMSE score**  **/30** | 28.12 (1.55) | 28.94 (1.34) | 27.67 (1.53) | 28.84 (1.23) | p = 0.20 |

**Table S5. Demographic Characteristics of participants across AT CSF subsample.**

|  | **A+T+**  **(n = 14)** | **A+T-**  **(n = 3)** | **A-T+**  **(n = 2)** | **A-T-**  **(n = 74)** | **Group differences** |
| --- | --- | --- | --- | --- | --- |
| **Age at baseline, years** | 64.41 (5.18) | 68.29 (2.63) | 65.67 (4.80) | 62.69 (4.27) | p = 0.09 |
| **Age at CSF visit, years** | 66.57 (5.58) | 71.37 (1.72) | 69.73 (4.69) | 64.81 (4.50) | p = 0.03 |
| **Sex, F, n (%)** | 6 (43) | 2 (67) | 0 (0) | 57 (77) | p = 0.005 ^c^ |
| **Education, years** | 13.29 (2.89) | 14.00 (3.61) | 18.50 (2.12) | 15.08 (2.78) | p = 0.03 |
| ***APOE* ε4 carriers, n (%)** | 13 (93) | 0 (0) | 1 (50) | 25 (34) | p < 0.001 ^a,c^ |
| **Aβ_42/40_** | 0.05 (0.01) | 0.05 (0.02) | 0.09 (0.01) | 0.10 (0.01) | p < 0.001 ^c,e^ |
| **pTau217**  **(pg/ml)** | 653.29 (119.29) | 310.13 (33.25) | 434.00 (8.59) | 188.92 (79.49) | p < 0.001 ^c^ |
| **MoCA score**  **/30** | 28.00 (1.36) | 28.33 (1.53) | 29.50 (0.71) | 28.03 (1.49) | p = 0.47 |
| **RBANS global score** | 97.07 (11.17) | 105.67 (6.66) | 100.00 (2.83) | 102.64 (9.52) | p = 0.19 |
| **MMSE score**  **/30** | 28.14 (1.92) | 29.33 (1.15) | 29.50 (0.71) | 28.85 (1.18) | p = 0.49 |

**Table S6. Demographic Characteristics of participants across AT PET subsample.**

|  | **A+T+**  **(n = 3)** | **A+T-**  **(n = 28)** | **A-T+**  **(n = 1)** | **A-T-**  **(n = 61)** | **Group differences** |
| --- | --- | --- | --- | --- | --- |
| **Age at baseline, years** | 64.34 (4.64) | 63.63 (4.41) | 62.90 | 62.94 (4.59) | p = 0.72 |
| **Age at PET, years** | 69.53 (4.09) | 67.38 (4.83) | 64.42 | 66.71 (4.62) | p = 0.47 |
| **Sex, F, n (%)** | 2 (67) | 18 (64) | 1(100) | 44 (72) | p = 0.76 |
| **Education, years** | 14.67 (3.79) | 13.82 (2.25) | 11.00 | 15.39 (3.01) | p = 0.05^e^ |
| ***APOE* ε4 carriers, n (%)** | 3 (100) | 16 (57) | 1(100) | 19 (31) | p = 0.007 ^c,e^ |
| **Global Aβ SUVR** | 2.14 (0.30) | 1.51 (0.27) | 1.25 | 1.16 (0.06) | p < 0.001 ^c,e^ |
| **Temporal meta-ROI SUVR** | 1.52 (0.09) | 1.17 (0.07) | 1.55 | 1.13 (0.07) | p < 0.001^a,c,e^ |
| **MoCA score**  **/30** | 28.67 (1.53) | 28.14 (1.60) | 28.00 | 28.00 (1.41) | p = 0.62 |
| **RBANS global score** | 93.00 (12.12) | 101.43 (10.21) | 86.00 | 102.93 (9.22) | p = 0.23 |
| **MMSE score/30** | 27.33 (2.31) | 28.86 (1.38) | 29.00 | 28.80 (1.24) | p = 0.40 |

**Table (S4-6)** Data presented as mean (standard deviation), except for categorical variables where the count and percentage are presented. Fisher or Kruskal-Wallis test was performed between the A/T groups from the subsample (n = 93) and p values are reported on the right column. If a significant group difference (p<0.05) was found post-hoc p values are reported (^a^ difference between A+T+ and A+T-; ^b^ difference between A+T+ and A-T+; ^c^ difference between A+T+ and A-T-; ^d^ difference between A+T- and A-T+; ^e^ A+T- and A-T-; ^f^ A-T+ and A-T-). Age at baseline and at biomarker measurement are presented; RBANS values are shown at baseline. MoCA scores were collected at entry into the program. MoCA score was missing for one participant in the plasma and CSF A-T- group. Abbreviations: MoCA = Montreal Cognitive Assessment; F = female; APOE = apolipoprotein E; SUVR = Standardized Uptake Value Ratio; RBANS = Repeatable Battery for the Assessment of Neuropsychological Status.

**Table S7. Diagnostic accuracy of plasma p-tau217 in predicting MCI**

| \| **Plasma p-tau217** \| \| \| \| \| \| --- \| --- \| --- \| --- \| --- \| \| **Cutoff** \| **Sensitivity,%** \| **Specificity,%** \| **NPV,%** \| **PPV,%** \| \| 1.015 \| 100 \| 1.56 \| 100 \| 31.52 \| \| 1.075 \| 100 \| 3.12 \| 100 \| 31.87 \| \| 1.15 \| 100 \| 4.69 \| 100 \| 32.22 \| \| 1.235 \| 100 \| 6.25 \| 100 \| 32.58 \| \| 1.265 \| 100 \| 7.81 \| 100 \| 32.95 \| \| 1.355 \| 96.55 \| 7.81 \| 83.33 \| 32.18 \| \| 1.44 \| 96.55 \| 9.38 \| 85.71 \| 32.56 \| \| 1.47 \| 96.55 \| 10.94 \| 87.5 \| 32.94 \| \| 1.495 \| 96.55 \| 12.5 \| 88.89 \| 33.33 \| \| 1.505 \| 96.55 \| 14.06 \| 90 \| 33.73 \| \| 1.555 \| 93.1 \| 14.06 \| 81.82 \| 32.93 \| \| 1.625 \| 93.1 \| 15.62 \| 83.33 \| 33.33 \| \| 1.66 \| 93.1 \| 17.19 \| 84.62 \| 33.75 \| \| 1.685 \| 89.66 \| 17.19 \| 78.57 \| 32.91 \| \| 1.705 \| 89.66 \| 18.75 \| 80 \| 33.33 \| \| 1.72 \| 89.66 \| 21.88 \| 82.35 \| 34.21 \| \| 1.735 \| 89.66 \| 23.44 \| 83.33 \| 34.67 \| \| 1.745 \| 89.66 \| 25 \| 84.21 \| 35.14 \| \| 1.76 \| 89.66 \| 26.56 \| 85 \| 35.62 \| \| 1.78 \| 86.21 \| 28.12 \| 81.82 \| 35.21 \| \| 1.8 \| 86.21 \| 29.69 \| 82.61 \| 35.71 \| \| 1.815 \| 86.21 \| 32.81 \| 84 \| 36.76 \| \| 1.825 \| 86.21 \| 34.38 \| 84.62 \| 37.31 \| \| 1.845 \| 82.76 \| 35.94 \| 82.14 \| 36.92 \| \| 1.87 \| 82.76 \| 39.06 \| 83.33 \| 38.1 \| \| 1.895 \| 82.76 \| 40.62 \| 83.87 \| 38.71 \| \| 1.92 \| 79.31 \| 45.31 \| 82.86 \| 39.66 \| \| 1.94 \| 79.31 \| 46.88 \| 83.33 \| 40.35 \| \| 1.96 \| 75.86 \| 46.88 \| 81.08 \| 39.29 \| \| 1.98 \| 75.86 \| 48.44 \| 81.58 \| 40 \| \| 1.995 \| 75.86 \| 50 \| 82.05 \| 40.74 \| \| 2.005 \| 75.86 \| 51.56 \| 82.5 \| 41.51 \| \| 2.015 \| 75.86 \| 53.12 \| 82.93 \| 42.31 \| \| 2.065 \| 72.41 \| 53.12 \| 80.95 \| 41.18 \| \| 2.13 \| 68.97 \| 53.12 \| 79.07 \| 40 \| \| 2.18 \| 68.97 \| 54.69 \| 79.55 \| 40.82 \| \| 2.235 \| 68.97 \| 57.81 \| 80.43 \| 42.55 \| \| 2.265 \| 68.97 \| 59.38 \| 80.85 \| 43.48 \| \| 2.28 \| 68.97 \| 62.5 \| 81.63 \| 45.45 \| \| 2.295 \| 68.97 \| 64.06 \| 82 \| 46.51 \| \| 2.315 \| 68.97 \| 65.62 \| 82.35 \| 47.62 \| \| 2.335 \| 65.52 \| 65.62 \| 80.77 \| 46.34 \| \| 2.345 \| 65.52 \| 67.19 \| 81.13 \| 47.5 \| \| 2.365 \| 65.52 \| 68.75 \| 81.48 \| 48.72 \| \| 2.395 \| 65.52 \| 70.31 \| 81.82 \| 50 \| \| 2.415 \| 65.52 \| 71.88 \| 82.14 \| 51.35 \| \| 2.445 \| 65.52 \| 73.44 \| 82.46 \| 52.78 \| \| 2.48 \| 65.52 \| 75 \| 82.76 \| 54.29 \| \| 2.505 \| 65.52 \| 76.56 \| 83.05 \| 55.88 \| \| 2.56 \| 65.52 \| 78.12 \| 83.33 \| 57.58 \| \| 2.605 \| 65.52 \| 79.69 \| 83.61 \| 59.38 \| \| 2.635 \| 65.52 \| 81.25 \| 83.87 \| 61.29 \| \| 2.685 \| 65.52 \| 82.81 \| 84.13 \| 63.33 \| \| 2.715 \| 62.07 \| 82.81 \| 82.81 \| 62.07 \| \| 2.735 \| 58.62 \| 82.81 \| 81.54 \| 60.71 \| \| 2.775 \| 51.72 \| 82.81 \| 79.1 \| 57.69 \| \| 2.845 \| 51.72 \| 84.38 \| 79.41 \| 60 \| \| 2.97 \| 51.72 \| 85.94 \| 79.71 \| 62.5 \| \| 3.105 \| 51.72 \| 87.5 \| 80 \| 65.22 \| \| 3.175 \| 51.72 \| 89.06 \| 80.28 \| 68.18 \| \| 3.21 \| 51.72 \| 90.62 \| 80.56 \| 71.43 \| \| 3.265 \| 48.28 \| 90.62 \| 79.45 \| 70 \| \| 3.32 \| 48.28 \| 92.19 \| 79.73 \| 73.68 \| \| 3.38 \| 48.28 \| 93.75 \| 80 \| 77.78 \| \| 3.485 \| 44.83 \| 93.75 \| 78.95 \| 76.47 \| \| 3.565 \| 44.83 \| 95.31 \| 79.22 \| 81.25 \| \| 3.595 \| 44.83 \| 96.88 \| 79.49 \| 86.67 \| \| 3.615 \| 41.38 \| 96.88 \| 78.48 \| 85.71 \| \| 3.7 \| 37.93 \| 96.88 \| 77.5 \| 84.62 \| \| **3.81** \| **34.48** \| **96.88** \| **76.54** \| **83.33** \| \| 4.27 \| 34.48 \| 98.44 \| 76.83 \| 90.91 \| \| 4.745 \| 31.03 \| 100 \| 76.19 \| 100 \| \| 4.815 \| 24.14 \| 100 \| 74.42 \| 100 \| \| 5.03 \| 20.69 \| 100 \| 73.56 \| 100 \| \| 5.26 \| 17.24 \| 100 \| 72.73 \| 100 \| \| 5.38 \| 13.79 \| 100 \| 71.91 \| 100 \| \| 5.645 \| 10.34 \| 100 \| 71.11 \| 100 \| \| 6.285 \| 6.9 \| 100 \| 70.33 \| 100 \| \| 6.915 \| 3.45 \| 100 \| 69.57 \| 100 \| |
| --- | --- | --- | --- | --- | --- | --- | --- | --- | --- | --- | --- | --- | --- | --- | --- | --- | --- | --- | --- | --- | --- | --- | --- | --- | --- | --- | --- | --- | --- | --- | --- | --- | --- | --- | --- | --- | --- | --- | --- | --- | --- | --- | --- | --- | --- | --- | --- | --- | --- | --- | --- | --- | --- | --- | --- | --- | --- | --- | --- | --- | --- | --- | --- | --- | --- | --- | --- | --- | --- | --- | --- | --- | --- | --- | --- | --- | --- | --- | --- | --- | --- | --- | --- | --- | --- | --- | --- | --- | --- | --- | --- | --- | --- | --- | --- | --- | --- | --- | --- | --- | --- | --- | --- | --- | --- | --- | --- | --- | --- | --- | --- | --- | --- | --- | --- | --- | --- | --- | --- | --- | --- | --- | --- | --- | --- | --- | --- | --- | --- | --- | --- | --- | --- | --- | --- | --- | --- | --- | --- | --- | --- | --- | --- | --- | --- | --- | --- | --- | --- | --- | --- | --- | --- | --- | --- | --- | --- | --- | --- | --- | --- | --- | --- | --- | --- | --- | --- | --- | --- | --- | --- | --- | --- | --- | --- | --- | --- | --- | --- | --- | --- | --- | --- | --- | --- | --- | --- | --- | --- | --- | --- | --- | --- | --- | --- | --- | --- | --- | --- | --- | --- | --- | --- | --- | --- | --- | --- | --- | --- | --- | --- | --- | --- | --- | --- | --- | --- | --- | --- | --- | --- | --- | --- | --- | --- | --- | --- | --- | --- | --- | --- | --- | --- | --- | --- | --- | --- | --- | --- | --- | --- | --- | --- | --- | --- | --- | --- | --- | --- | --- | --- | --- | --- | --- | --- | --- | --- | --- | --- | --- | --- | --- | --- | --- | --- | --- | --- | --- | --- | --- | --- | --- | --- | --- | --- | --- | --- | --- | --- | --- | --- | --- | --- | --- | --- | --- | --- | --- | --- | --- | --- | --- | --- | --- | --- | --- | --- | --- | --- | --- | --- | --- | --- | --- | --- | --- | --- | --- | --- | --- | --- | --- | --- | --- | --- | --- | --- | --- | --- | --- | --- | --- | --- | --- | --- | --- | --- | --- | --- | --- | --- | --- | --- | --- | --- | --- | --- | --- | --- | --- | --- | --- | --- | --- | --- | --- | --- | --- | --- | --- | --- | --- | --- | --- | --- | --- | --- | --- | --- | --- | --- | --- | --- | --- | --- | --- | --- | --- | --- | --- | --- | --- | --- | --- | --- | --- | --- | --- | --- | --- | --- | --- | --- | --- | --- | --- | --- | --- | --- | --- | --- | --- | --- | --- | --- | --- | --- | --- | --- | --- | --- | --- | --- | --- | --- |

**Table S8. Diagnostic accuracy of plasma Aβ_42/40_ in predicting MCI**

**Plasma Aβ_42/40_**

**Cutoff Sensitivity,% Specificity,% NPV,% PPV,%**

| 0.125 | 100 | 1.56 | 100 | 31.52 |
| --- | --- | --- | --- | --- |
| 0.1235 | 96.55 | 1.56 | 50 | 30.77 |
| 0.1225 | 93.1 | 1.56 | 33.33 | 30 |
| 0.121 | 93.1 | 3.12 | 50 | 30.34 |
| 0.1195 | 93.1 | 4.69 | 60 | 30.68 |
| 0.1175 | 93.1 | 6.25 | 66.67 | 31.03 |
| 0.115 | 93.1 | 7.81 | 71.43 | 31.4 |
| 0.113 | 93.1 | 9.38 | 75 | 31.76 |
| 0.111 | 89.66 | 9.38 | 66.67 | 30.95 |
| 0.1095 | 89.66 | 10.94 | 70 | 31.33 |
| 0.1085 | 89.66 | 12.5 | 72.73 | 31.71 |
| 0.1075 | 89.66 | 15.62 | 76.92 | 32.5 |
| 0.1065 | 89.66 | 17.19 | 78.57 | 32.91 |
| 0.1055 | 89.66 | 20.31 | 81.25 | 33.77 |
| 0.1045 | 86.21 | 23.44 | 78.95 | 33.78 |
| 0.103 | 79.31 | 28.12 | 75 | 33.33 |
| 0.1015 | 79.31 | 32.81 | 77.78 | 34.85 |
| 0.1 | 75.86 | 35.94 | 76.67 | 34.92 |
| 0.0985 | 75.86 | 40.62 | 78.79 | 36.67 |
| 0.0975 | 75.86 | 45.31 | 80.56 | 38.6 |
| 0.0965 | 75.86 | 53.12 | 82.93 | 42.31 |
| 0.0955 | 75.86 | 54.69 | 83.33 | 43.14 |
| 0.0945 | 75.86 | 57.81 | 84.09 | 44.9 |
| 0.0935 | 72.41 | 59.38 | 82.61 | 44.68 |
| 0.0925 | 72.41 | 64.06 | 83.67 | 47.73 |
| 0.0915 | 68.97 | 65.62 | 82.35 | 47.62 |
| **0.0905** | **65.52** | **65.62** | **80.77** | **46.34** |
| 0.0895 | 65.52 | 68.75 | 81.48 | 48.72 |
| 0.0885 | 62.07 | 70.31 | 80.36 | 48.65 |
| 0.087 | 55.17 | 71.88 | 77.97 | 47.06 |
| 0.0855 | 51.72 | 75 | 77.42 | 48.39 |
| 0.0845 | 48.28 | 78.12 | 76.92 | 50 |
| 0.0835 | 37.93 | 81.25 | 74.29 | 47.83 |
| 0.082 | 34.48 | 84.38 | 73.97 | 50 |
| 0.0805 | 27.59 | 85.94 | 72.37 | 47.06 |
| 0.0795 | 24.14 | 85.94 | 71.43 | 43.75 |
| 0.0785 | 24.14 | 87.5 | 71.79 | 46.67 |
| 0.0775 | 20.69 | 89.06 | 71.25 | 46.15 |
| 0.0765 | 20.69 | 90.62 | 71.6 | 50 |
| 0.0755 | 20.69 | 92.19 | 71.95 | 54.55 |
| 0.074 | 17.24 | 95.31 | 71.76 | 62.5 |
| 0.0725 | 13.79 | 95.31 | 70.93 | 57.14 |
| 0.0715 | 10.34 | 95.31 | 70.11 | 50 |
| 0.0695 | 3.45 | 96.88 | 68.89 | 33.33 |
| 0.067 | 0 | 96.88 | 68.13 | 0 |
| 0.0485 | 0 | 98.44 | 68.48 | 0 |

**Table S9. Diagnostic accuracy of CSF p-tau217 in predicting MCI**

| **CSF p-tau217** | | | | |
| --- | --- | --- | --- | --- |
| **Cutoff** | **Sensitivity,%** | **Specificity,%** | **NPV,%** | **PPV,%** |
| 76.905 | 100 | 1.56 | 100 | 31.52 |
| 82.545 | 100 | 3.12 | 100 | 31.87 |
| 84.945 | 100 | 4.69 | 100 | 32.22 |
| 86.87 | 100 | 6.25 | 100 | 32.58 |
| 87.895 | 100 | 7.81 | 100 | 32.95 |
| 89.52 | 100 | 9.38 | 100 | 33.33 |
| 91.465 | 100 | 10.94 | 100 | 33.72 |
| 92.845 | 100 | 12.5 | 100 | 34.12 |
| 95.275 | 100 | 14.06 | 100 | 34.52 |
| 98.935 | 96.55 | 14.06 | 90 | 33.73 |
| 101.08 | 96.55 | 15.62 | 90.91 | 34.15 |
| 101.48 | 96.55 | 17.19 | 91.67 | 34.57 |
| 102.105 | 96.55 | 18.75 | 92.31 | 35 |
| 106.965 | 96.55 | 20.31 | 92.86 | 35.44 |
| 112.345 | 93.1 | 20.31 | 86.67 | 34.62 |
| 114.815 | 93.1 | 21.88 | 87.5 | 35.06 |
| 120.135 | 93.1 | 23.44 | 88.24 | 35.53 |
| 125.145 | 93.1 | 25 | 88.89 | 36 |
| 127.915 | 93.1 | 26.56 | 89.47 | 36.49 |
| 131.105 | 93.1 | 28.12 | 90 | 36.99 |
| 133.045 | 93.1 | 29.69 | 90.48 | 37.5 |
| 133.65 | 93.1 | 31.25 | 90.91 | 38.03 |
| 135.37 | 89.66 | 31.25 | 86.96 | 37.14 |
| 139.7 | 89.66 | 32.81 | 87.5 | 37.68 |
| 145.89 | 89.66 | 34.38 | 88 | 38.24 |
| 149.18 | 89.66 | 35.94 | 88.46 | 38.81 |
| 151.57 | 89.66 | 37.5 | 88.89 | 39.39 |
| 154.39 | 89.66 | 39.06 | 89.29 | 40 |
| 155.355 | 89.66 | 40.62 | 89.66 | 40.62 |
| 156.13 | 89.66 | 42.19 | 90 | 41.27 |
| 158.265 | 86.21 | 42.19 | 87.1 | 40.32 |
| 161.155 | 86.21 | 45.31 | 87.88 | 41.67 |
| 163.33 | 86.21 | 46.88 | 88.24 | 42.37 |
| 166.215 | 86.21 | 48.44 | 88.57 | 43.1 |
| 170.11 | 86.21 | 50 | 88.89 | 43.86 |
| 173.11 | 86.21 | 51.56 | 89.19 | 44.64 |
| 176.05 | 86.21 | 53.12 | 89.47 | 45.45 |
| 180.57 | 86.21 | 54.69 | 89.74 | 46.3 |
| 184.54 | 86.21 | 56.25 | 90 | 47.17 |
| 186.625 | 82.76 | 56.25 | 87.8 | 46.15 |
| 188.45 | 82.76 | 57.81 | 88.1 | 47.06 |
| 196.42 | 82.76 | 59.38 | 88.37 | 48 |
| 206.71 | 79.31 | 59.38 | 86.36 | 46.94 |
| 213.51 | 79.31 | 60.94 | 86.67 | 47.92 |
| 217.335 | 79.31 | 62.5 | 86.96 | 48.94 |
| 218.865 | 79.31 | 64.06 | 87.23 | 50 |
| 219.99 | 79.31 | 65.62 | 87.5 | 51.11 |
| 220.355 | 79.31 | 67.19 | 87.76 | 52.27 |
| 223.14 | 75.86 | 67.19 | 86 | 51.16 |
| 225.735 | 75.86 | 68.75 | 86.27 | 52.38 |
| 226.4 | 75.86 | 70.31 | 86.54 | 53.66 |
| 228.635 | 75.86 | 71.88 | 86.79 | 55 |
| 230.28 | 72.41 | 71.88 | 85.19 | 53.85 |
| 234.345 | 72.41 | 73.44 | 85.45 | 55.26 |
| 238.52 | 72.41 | 75 | 85.71 | 56.76 |
| 240.045 | 72.41 | 76.56 | 85.96 | 58.33 |
| 243.25 | 72.41 | 78.12 | 86.21 | 60 |
| 249.11 | 72.41 | 79.69 | 86.44 | 61.76 |
| 256.365 | 72.41 | 81.25 | 86.67 | 63.64 |
| 261.555 | 72.41 | 82.81 | 86.89 | 65.62 |
| 272.12 | 68.97 | 82.81 | 85.48 | 64.52 |
| 281.975 | 65.52 | 82.81 | 84.13 | 63.33 |
| 285.095 | 65.52 | 84.38 | 84.38 | 65.52 |
| 287.205 | 65.52 | 85.94 | 84.62 | 67.86 |
| 293.655 | 62.07 | 85.94 | 83.33 | 66.67 |
| 306.445 | 58.62 | 85.94 | 82.09 | 65.38 |
| 316.54 | 55.17 | 85.94 | 80.88 | 64 |
| 320.625 | 55.17 | 87.5 | 81.16 | 66.67 |
| 323.37 | 55.17 | 89.06 | 81.43 | 69.57 |
| 335.92 | 55.17 | 90.62 | 81.69 | 72.73 |
| 346.785 | 51.72 | 90.62 | 80.56 | 71.43 |
| 347.54 | 51.72 | 92.19 | 80.82 | 75 |
| 351.04 | 48.28 | 92.19 | 79.73 | 73.68 |
| 357.425 | 48.28 | 93.75 | 80 | 77.78 |
| 362.57 | 44.83 | 93.75 | 78.95 | 76.47 |
| **396.255** | **44.83** | **95.31** | **79.22** | **81.25** |
| 433.995 | 44.83 | 96.88 | 79.49 | 86.67 |
| 498.345 | 41.38 | 96.88 | 78.48 | 85.71 |
| 560.94 | 41.38 | 98.44 | 78.75 | 92.31 |
| 569.905 | 37.93 | 98.44 | 77.78 | 91.67 |
| 579.18 | 34.48 | 98.44 | 76.83 | 90.91 |
| 589.27 | 31.03 | 98.44 | 75.9 | 90 |
| 597.845 | 27.59 | 98.44 | 75 | 88.89 |
| 605.82 | 24.14 | 98.44 | 74.12 | 87.5 |
| 611.95 | 20.69 | 98.44 | 73.26 | 85.71 |
| 626.95 | 17.24 | 98.44 | 72.41 | 83.33 |
| 647.61 | 13.79 | 98.44 | 71.59 | 80 |
| 672.005 | 10.34 | 98.44 | 70.79 | 75 |
| 693.45 | 6.9 | 98.44 | 70 | 66.67 |
| 719.715 | 3.45 | 98.44 | 69.23 | 50 |
| 882.04 | 3.45 | 100 | 69.57 | 100 |

**Table S10. Diagnostic accuracy of CSF Aβ_42/40_ in predicting MCI**

| **CSF Aβ_42/40_** | | | | |
| --- | --- | --- | --- | --- |
| **Cutoff** | **Sensitivity,%** | **Specificity,%** | **NPV,%** | **PPV,%** |
| 0.11755925 | 100 | 1.56 | 100 | 31.52 |
| 0.11629457 | 100 | 3.12 | 100 | 31.87 |
| 0.11550666 | 100 | 4.69 | 100 | 32.22 |
| 0.11520654 | 100 | 6.25 | 100 | 32.58 |
| 0.11467891 | 96.55 | 6.25 | 80 | 31.82 |
| 0.11431674 | 93.1 | 6.25 | 66.67 | 31.03 |
| 0.11387694 | 93.1 | 7.81 | 71.43 | 31.4 |
| 0.11314396 | 93.1 | 9.38 | 75 | 31.76 |
| 0.11241106 | 93.1 | 10.94 | 77.78 | 32.14 |
| 0.1115638 | 93.1 | 12.5 | 80 | 32.53 |
| 0.11078507 | 89.66 | 12.5 | 72.73 | 31.71 |
| 0.11041892 | 89.66 | 14.06 | 75 | 32.1 |
| 0.1103296 | 89.66 | 15.62 | 76.92 | 32.5 |
| 0.11002579 | 89.66 | 17.19 | 78.57 | 32.91 |
| 0.1097942 | 89.66 | 18.75 | 80 | 33.33 |
| 0.1094816 | 89.66 | 20.31 | 81.25 | 33.77 |
| 0.10891789 | 89.66 | 21.88 | 82.35 | 34.21 |
| 0.10865698 | 86.21 | 21.88 | 77.78 | 33.33 |
| 0.10836479 | 86.21 | 23.44 | 78.95 | 33.78 |
| 0.10793686 | 86.21 | 25 | 80 | 34.25 |
| 0.10771086 | 82.76 | 25 | 76.19 | 33.33 |
| 0.10761779 | 82.76 | 26.56 | 77.27 | 33.8 |
| 0.10757785 | 79.31 | 26.56 | 73.91 | 32.86 |
| 0.10733048 | 75.86 | 26.56 | 70.83 | 31.88 |
| 0.1071193 | 75.86 | 28.12 | 72 | 32.35 |
| 0.10698218 | 75.86 | 29.69 | 73.08 | 32.84 |
| 0.106841 | 75.86 | 31.25 | 74.07 | 33.33 |
| 0.10667824 | 75.86 | 32.81 | 75 | 33.85 |
| 0.10597734 | 75.86 | 34.38 | 75.86 | 34.38 |
| 0.10542111 | 75.86 | 35.94 | 76.67 | 34.92 |
| 0.10515465 | 75.86 | 37.5 | 77.42 | 35.48 |
| 0.10487495 | 75.86 | 39.06 | 78.12 | 36.07 |
| 0.10473536 | 72.41 | 39.06 | 75.76 | 35 |
| 0.10460357 | 72.41 | 40.62 | 76.47 | 35.59 |
| 0.10387171 | 68.97 | 40.62 | 74.29 | 34.48 |
| 0.10312531 | 68.97 | 42.19 | 75 | 35.09 |
| 0.10245297 | 68.97 | 43.75 | 75.68 | 35.71 |
| 0.10177143 | 68.97 | 45.31 | 76.32 | 36.36 |
| 0.10163765 | 68.97 | 46.88 | 76.92 | 37.04 |
| 0.10123674 | 68.97 | 48.44 | 77.5 | 37.74 |
| 0.10078462 | 68.97 | 50 | 78.05 | 38.46 |
| 0.10052623 | 68.97 | 51.56 | 78.57 | 39.22 |
| 0.09991946 | 68.97 | 53.12 | 79.07 | 40 |
| 0.0993973 | 68.97 | 54.69 | 79.55 | 40.82 |
| 0.09935234 | 65.52 | 54.69 | 77.78 | 39.58 |
| 0.09921656 | 65.52 | 56.25 | 78.26 | 40.43 |
| 0.09807373 | 62.07 | 56.25 | 76.6 | 39.13 |
| 0.09658938 | 62.07 | 57.81 | 77.08 | 40 |
| 0.09594203 | 62.07 | 59.38 | 77.55 | 40.91 |
| 0.09572916 | 58.62 | 59.38 | 76 | 39.53 |
| 0.09561533 | 58.62 | 60.94 | 76.47 | 40.48 |
| 0.09544816 | 58.62 | 62.5 | 76.92 | 41.46 |
| 0.09527615 | 58.62 | 64.06 | 77.36 | 42.5 |
| 0.09475347 | 58.62 | 65.62 | 77.78 | 43.59 |
| 0.09430784 | 58.62 | 67.19 | 78.18 | 44.74 |
| 0.0941041 | 58.62 | 68.75 | 78.57 | 45.95 |
| 0.09372267 | 58.62 | 70.31 | 78.95 | 47.22 |
| 0.09298958 | 58.62 | 71.88 | 79.31 | 48.57 |
| 0.09123886 | 58.62 | 73.44 | 79.66 | 50 |
| 0.08992333 | 58.62 | 75 | 80 | 51.52 |
| 0.0897631 | 58.62 | 76.56 | 80.33 | 53.12 |
| 0.08952656 | 58.62 | 78.12 | 80.65 | 54.84 |
| 0.08891739 | 58.62 | 79.69 | 80.95 | 56.67 |
| 0.08831979 | 55.17 | 79.69 | 79.69 | 55.17 |
| 0.08789368 | 51.72 | 79.69 | 78.46 | 53.57 |
| 0.08757762 | 51.72 | 81.25 | 78.79 | 55.56 |
| 0.08747697 | 48.28 | 81.25 | 77.61 | 53.85 |
| 0.08688775 | 48.28 | 82.81 | 77.94 | 56 |
| 0.08346913 | 48.28 | 84.38 | 78.26 | 58.33 |
| 0.07954653 | 48.28 | 85.94 | 78.57 | 60.87 |
| 0.07816239 | 48.28 | 87.5 | 78.87 | 63.64 |
| 0.07781973 | 44.83 | 87.5 | 77.78 | 61.9 |
| 0.07774518 | 44.83 | 89.06 | 78.08 | 65 |
| 0.0770715 | 44.83 | 90.62 | 78.38 | 68.42 |
| 0.07589199 | 44.83 | 92.19 | 78.67 | 72.22 |
| **0.07027368** | **44.83** | **93.75** | **78.95** | **76.47** |
| 0.06507624 | 44.83 | 95.31 | 79.22 | 81.25 |
| 0.06481599 | 41.38 | 95.31 | 78.21 | 80 |
| 0.06305217 | 41.38 | 96.88 | 78.48 | 85.71 |
| 0.05935628 | 37.93 | 96.88 | 77.5 | 84.62 |
| 0.05722767 | 34.48 | 96.88 | 76.54 | 83.33 |
| 0.05699247 | 31.03 | 96.88 | 75.61 | 81.82 |
| 0.05495576 | 27.59 | 96.88 | 74.7 | 80 |
| 0.05300843 | 24.14 | 96.88 | 73.81 | 77.78 |
| 0.0522687 | 20.69 | 96.88 | 72.94 | 75 |
| 0.04782442 | 17.24 | 96.88 | 72.09 | 71.43 |
| 0.04401495 | 13.79 | 96.88 | 71.26 | 66.67 |
| 0.04304896 | 10.34 | 96.88 | 70.45 | 60 |
| 0.04109527 | 6.9 | 96.88 | 69.66 | 50 |
| 0.03958742 | 3.45 | 96.88 | 68.89 | 33.33 |
| 0.03815797 | 3.45 | 98.44 | 69.23 | 50 |
| 0.03708082 | 0 | 98.44 | 68.48 | 0 |

**Table S11. Diagnostic accuracy of meta-ROI tau-PET in predicting MCI**

| **Meta-ROI tau-PET** | | | | |
| --- | --- | --- | --- | --- |
| **Cutoff** | **Sensitivity,%** | **Specificity,%** | **NPV,%** | **PPV,%** |
| 0.93387917 | 100 | 1.56 | 100 | 31.52 |
| 1.02888333 | 100 | 3.12 | 100 | 31.87 |
| 1.03296667 | 100 | 4.69 | 100 | 32.22 |
| 1.04064583 | 100 | 6.25 | 100 | 32.58 |
| 1.04680833 | 96.55 | 6.25 | 80 | 31.82 |
| 1.05005833 | 96.55 | 7.81 | 83.33 | 32.18 |
| 1.05174583 | 96.55 | 9.38 | 85.71 | 32.56 |
| 1.05585833 | 96.55 | 10.94 | 87.5 | 32.94 |
| 1.059225 | 96.55 | 12.5 | 88.89 | 33.33 |
| 1.06004167 | 96.55 | 14.06 | 90 | 33.73 |
| 1.06235833 | 96.55 | 15.62 | 90.91 | 34.15 |
| 1.06590417 | 96.55 | 17.19 | 91.67 | 34.57 |
| 1.07028333 | 96.55 | 18.75 | 92.31 | 35 |
| 1.07317083 | 96.55 | 20.31 | 92.86 | 35.44 |
| 1.07369167 | 96.55 | 21.88 | 93.33 | 35.9 |
| 1.07482083 | 96.55 | 23.44 | 93.75 | 36.36 |
| 1.0761625 | 96.55 | 25 | 94.12 | 36.84 |
| 1.07802917 | 96.55 | 26.56 | 94.44 | 37.33 |
| 1.08136667 | 93.1 | 26.56 | 89.47 | 36.49 |
| 1.08465 | 93.1 | 28.12 | 90 | 36.99 |
| 1.0876125 | 93.1 | 29.69 | 90.48 | 37.5 |
| 1.091425 | 93.1 | 31.25 | 90.91 | 38.03 |
| 1.09397083 | 93.1 | 32.81 | 91.3 | 38.57 |
| 1.09449583 | 93.1 | 34.38 | 91.67 | 39.13 |
| 1.0954875 | 89.66 | 34.38 | 88 | 38.24 |
| 1.0968375 | 89.66 | 35.94 | 88.46 | 38.81 |
| 1.09965417 | 86.21 | 35.94 | 85.19 | 37.88 |
| 1.1036625 | 82.76 | 35.94 | 82.14 | 36.92 |
| 1.105975 | 79.31 | 35.94 | 79.31 | 35.94 |
| 1.10824583 | 79.31 | 37.5 | 80 | 36.51 |
| 1.11210833 | 79.31 | 39.06 | 80.65 | 37.1 |
| 1.11415417 | 79.31 | 40.62 | 81.25 | 37.7 |
| 1.11581667 | 75.86 | 40.62 | 78.79 | 36.67 |
| 1.11866667 | 75.86 | 42.19 | 79.41 | 37.29 |
| 1.12019167 | 75.86 | 43.75 | 80 | 37.93 |
| 1.12160417 | 75.86 | 45.31 | 80.56 | 38.6 |
| 1.12307917 | 75.86 | 46.88 | 81.08 | 39.29 |
| 1.1238375 | 75.86 | 48.44 | 81.58 | 40 |
| 1.12503333 | 75.86 | 50 | 82.05 | 40.74 |
| 1.12792917 | 75.86 | 51.56 | 82.5 | 41.51 |
| 1.130375 | 75.86 | 53.12 | 82.93 | 42.31 |
| 1.13249167 | 72.41 | 53.12 | 80.95 | 41.18 |
| 1.13436667 | 72.41 | 54.69 | 81.4 | 42 |
| 1.1346 | 72.41 | 56.25 | 81.82 | 42.86 |
| 1.13725833 | 72.41 | 57.81 | 82.22 | 43.75 |
| 1.14160417 | 72.41 | 59.38 | 82.61 | 44.68 |
| 1.14356667 | 72.41 | 60.94 | 82.98 | 45.65 |
| 1.14482917 | 72.41 | 62.5 | 83.33 | 46.67 |
| 1.14882083 | 68.97 | 62.5 | 81.63 | 45.45 |
| 1.15248333 | 68.97 | 64.06 | 82 | 46.51 |
| 1.15384583 | 68.97 | 65.62 | 82.35 | 47.62 |
| 1.1560875 | 68.97 | 67.19 | 82.69 | 48.78 |
| 1.15800833 | 68.97 | 68.75 | 83.02 | 50 |
| 1.16129167 | 68.97 | 70.31 | 83.33 | 51.28 |
| 1.1653 | 68.97 | 71.88 | 83.64 | 52.63 |
| 1.166475 | 68.97 | 73.44 | 83.93 | 54.05 |
| 1.16797083 | 65.52 | 73.44 | 82.46 | 52.78 |
| 1.16966667 | 65.52 | 75 | 82.76 | 54.29 |
| 1.17177917 | 65.52 | 76.56 | 83.05 | 55.88 |
| 1.17515417 | 65.52 | 78.12 | 83.33 | 57.58 |
| 1.17679167 | 62.07 | 78.12 | 81.97 | 56.25 |
| 1.17692083 | 58.62 | 78.12 | 80.65 | 54.84 |
| 1.178625 | 55.17 | 78.12 | 79.37 | 53.33 |
| 1.18093333 | 51.72 | 78.12 | 78.12 | 51.72 |
| 1.18207917 | 51.72 | 79.69 | 78.46 | 53.57 |
| 1.1826125 | 51.72 | 81.25 | 78.79 | 55.56 |
| 1.18456667 | 51.72 | 82.81 | 79.1 | 57.69 |
| 1.18687083 | 51.72 | 84.38 | 79.41 | 60 |
| 1.18985417 | 48.28 | 84.38 | 78.26 | 58.33 |
| 1.19388333 | 44.83 | 84.38 | 77.14 | 56.52 |
| 1.19658333 | 41.38 | 84.38 | 76.06 | 54.55 |
| 1.2024625 | 41.38 | 85.94 | 76.39 | 57.14 |
| 1.20722917 | 41.38 | 87.5 | 76.71 | 60 |
| 1.20845 | 37.93 | 87.5 | 75.68 | 57.89 |
| 1.214325 | 37.93 | 89.06 | 76 | 61.11 |
| 1.2193875 | 37.93 | 90.62 | 76.32 | 64.71 |
| 1.22206667 | 37.93 | 92.19 | 76.62 | 68.75 |
| 1.2259625 | 34.48 | 92.19 | 75.64 | 66.67 |
| 1.22948333 | 34.48 | 93.75 | 75.95 | 71.43 |
| 1.231575 | 31.03 | 93.75 | 75 | 69.23 |
| 1.23369583 | 27.59 | 93.75 | 74.07 | 66.67 |
| 1.2361 | 27.59 | 95.31 | 74.39 | 72.73 |
| 1.24079167 | 27.59 | 96.88 | 74.7 | 80 |
| 1.24540833 | 27.59 | 98.44 | 75 | 88.89 |
| 1.24978333 | 24.14 | 98.44 | 74.12 | 87.5 |
| 1.25456667 | 20.69 | 98.44 | 73.26 | 85.71 |
| 1.2602375 | 20.69 | 100 | 73.56 | 100 |
| **1.26750833** | **17.24** | **100** | **72.73** | **100** |
| 1.35673333 | 13.79 | 100 | 71.91 | 100 |
| 1.46423333 | 10.34 | 100 | 71.11 | 100 |
| 1.51714583 | 6.9 | 100 | 70.33 | 100 |
| 1.58541667 | 3.45 | 100 | 69.57 | 100 |

**Table S12. Diagnostic accuracy of Aβ-PET in predicting MCI**

**Aβ-PET**

| **Cutoff** | **Sensitivity,%** | **Specificity,%** | **NPV,%** | **PPV,%** |
| --- | --- | --- | --- | --- |
| 1.0291 | 100 | 1.56 | 100 | 31.52 |
| 1.0436 | 100 | 3.12 | 100 | 31.87 |
| 1.0659 | 100 | 4.69 | 100 | 32.22 |
| 1.0768 | 100 | 6.25 | 100 | 32.58 |
| 1.07995 | 96.55 | 6.25 | 80 | 31.82 |
| 1.0824 | 96.55 | 7.81 | 83.33 | 32.18 |
| 1.08475 | 96.55 | 9.38 | 85.71 | 32.56 |
| 1.0883 | 96.55 | 10.94 | 87.5 | 32.94 |
| 1.09195 | 96.55 | 12.5 | 88.89 | 33.33 |
| 1.09425 | 96.55 | 14.06 | 90 | 33.73 |
| 1.10765 | 96.55 | 15.62 | 90.91 | 34.15 |
| 1.11945 | 96.55 | 17.19 | 91.67 | 34.57 |
| 1.1206 | 96.55 | 18.75 | 92.31 | 35 |
| 1.12225 | 96.55 | 20.31 | 92.86 | 35.44 |
| 1.1269 | 96.55 | 21.88 | 93.33 | 35.9 |
| 1.13645 | 96.55 | 23.44 | 93.75 | 36.36 |
| 1.1441 | 93.1 | 23.44 | 88.24 | 35.53 |
| 1.1466 | 93.1 | 25 | 88.89 | 36 |
| 1.14845 | 93.1 | 26.56 | 89.47 | 36.49 |
| 1.14965 | 93.1 | 28.12 | 90 | 36.99 |
| 1.15115 | 93.1 | 29.69 | 90.48 | 37.5 |
| 1.1529 | 93.1 | 31.25 | 90.91 | 38.03 |
| 1.15345 | 93.1 | 32.81 | 91.3 | 38.57 |
| 1.1537 | 89.66 | 32.81 | 87.5 | 37.68 |
| 1.1546 | 86.21 | 32.81 | 84 | 36.76 |
| 1.1555 | 86.21 | 34.38 | 84.62 | 37.31 |
| 1.15585 | 86.21 | 35.94 | 85.19 | 37.88 |
| 1.15765 | 86.21 | 37.5 | 85.71 | 38.46 |
| 1.15945 | 86.21 | 39.06 | 86.21 | 39.06 |
| 1.15985 | 86.21 | 40.62 | 86.67 | 39.68 |
| 1.16065 | 86.21 | 42.19 | 87.1 | 40.32 |
| 1.162 | 86.21 | 43.75 | 87.5 | 40.98 |
| 1.16395 | 86.21 | 45.31 | 87.88 | 41.67 |
| 1.16525 | 86.21 | 46.88 | 88.24 | 42.37 |
| 1.1656 | 82.76 | 46.88 | 85.71 | 41.38 |
| 1.16625 | 82.76 | 48.44 | 86.11 | 42.11 |
| 1.17465 | 82.76 | 50 | 86.49 | 42.86 |
| 1.18295 | 82.76 | 51.56 | 86.84 | 43.64 |
| 1.187 | 82.76 | 53.12 | 87.18 | 44.44 |
| 1.19345 | 79.31 | 53.12 | 85 | 43.4 |
| 1.1976 | 79.31 | 54.69 | 85.37 | 44.23 |
| 1.2003 | 75.86 | 54.69 | 83.33 | 43.14 |
| 1.203 | 75.86 | 56.25 | 83.72 | 44 |
| 1.20465 | 75.86 | 57.81 | 84.09 | 44.9 |
| 1.20495 | 75.86 | 59.38 | 84.44 | 45.83 |
| 1.2052 | 72.41 | 59.38 | 82.61 | 44.68 |
| 1.2056 | 68.97 | 59.38 | 80.85 | 43.48 |
| 1.20705 | 65.52 | 59.38 | 79.17 | 42.22 |
| 1.2088 | 65.52 | 60.94 | 79.59 | 43.18 |
| 1.21065 | 62.07 | 60.94 | 78 | 41.86 |
| 1.21325 | 62.07 | 62.5 | 78.43 | 42.86 |
| 1.21585 | 62.07 | 64.06 | 78.85 | 43.9 |
| 1.21805 | 62.07 | 65.62 | 79.25 | 45 |
| 1.22365 | 62.07 | 67.19 | 79.63 | 46.15 |
| 1.23405 | 62.07 | 68.75 | 80 | 47.37 |
| 1.24325 | 62.07 | 70.31 | 80.36 | 48.65 |
| 1.24695 | 62.07 | 71.88 | 80.7 | 50 |
| 1.24755 | 62.07 | 73.44 | 81.03 | 51.43 |
| 1.2482 | 58.62 | 73.44 | 79.66 | 50 |
| 1.2493 | 58.62 | 75 | 80 | 51.52 |
| 1.25435 | 55.17 | 75 | 78.69 | 50 |
| **1.266** | **55.17** | **76.56** | **79.03** | **51.61** |
| 1.27355 | 51.72 | 76.56 | 77.78 | 50 |
| 1.27465 | 51.72 | 78.12 | 78.12 | 51.72 |
| 1.27925 | 51.72 | 79.69 | 78.46 | 53.57 |
| 1.2881 | 51.72 | 81.25 | 78.79 | 55.56 |
| 1.29385 | 48.28 | 81.25 | 77.61 | 53.85 |
| 1.29545 | 48.28 | 82.81 | 77.94 | 56 |
| 1.29725 | 48.28 | 84.38 | 78.26 | 58.33 |
| 1.3088 | 48.28 | 85.94 | 78.57 | 60.87 |
| 1.32835 | 48.28 | 87.5 | 78.87 | 63.64 |
| 1.349 | 44.83 | 87.5 | 77.78 | 61.9 |
| 1.3645 | 44.83 | 89.06 | 78.08 | 65 |
| 1.37455 | 44.83 | 90.62 | 78.38 | 68.42 |
| 1.38505 | 41.38 | 90.62 | 77.33 | 66.67 |
| 1.4041 | 41.38 | 92.19 | 77.63 | 70.59 |
| 1.4214 | 41.38 | 93.75 | 77.92 | 75 |
| 1.4348 | 41.38 | 95.31 | 78.21 | 80 |
| 1.4788 | 41.38 | 96.88 | 78.48 | 85.71 |
| 1.51825 | 37.93 | 96.88 | 77.5 | 84.62 |
| 1.56195 | 37.93 | 98.44 | 77.78 | 91.67 |
| 1.63095 | 34.48 | 98.44 | 76.83 | 90.91 |
| 1.66685 | 31.03 | 98.44 | 75.9 | 90 |
| 1.6735 | 27.59 | 98.44 | 75 | 88.89 |
| 1.70335 | 27.59 | 100 | 75.29 | 100 |
| 1.764 | 24.14 | 100 | 74.42 | 100 |
| 1.8415 | 20.69 | 100 | 73.56 | 100 |
| 1.89045 | 17.24 | 100 | 72.73 | 100 |
| 1.9434 | 13.79 | 100 | 71.91 | 100 |
| 2.11375 | 10.34 | 100 | 71.11 | 100 |
| 2.3088 | 6.9 | 100 | 70.33 | 100 |
| 2.39375 | 3.45 | 100 | 69.57 | 100 |

**Table S7-12.** Comparison estimates from ROC analyses of fluid biomarkers vs PET imaging in predicting MCI. The sensitivity, specificity, positive predictive values, and negative predictive values of the cutoffs used in the manuscript are presented in bold. Abbreviations: PPV = positive predictive value, NPV = negative predictive value.

**Figures**

**Figure S1. Flowchart of participants included** **in the study**. We included a total of 215 participants from plasma sample; 159 participants from the CSF sample; and 155 participants from PET sample in the main analyses. A total of 93 participants that have all biomarker measurements were included as a subsample. *Note*: One participant in the current study progressed to MCI after their baseline visit. While included in the study analyses, this participant is not considered as an active PREVENT-AD participant.

**
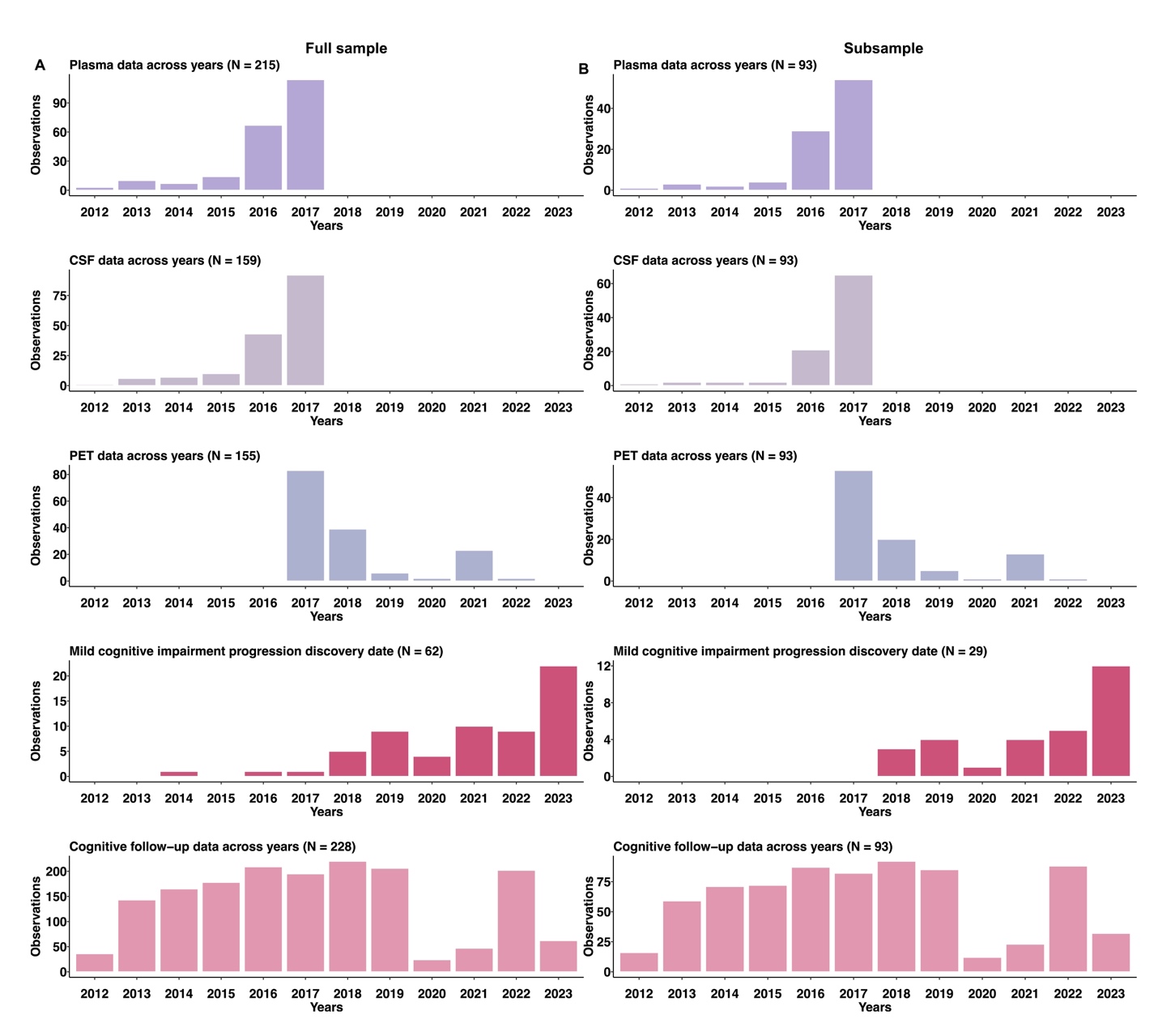
**

**Figure S2. Distribution of plasma, CSF, PET, and cognitive measurements.** Histograms illustrating the temporal distribution of assessments for plasma, CSF, and PET biomarkers, alongside the discovery dates of cognitive impairment progression, and longitudinal cognitive follow-up data across **A)** full sample and **B)** subsample.


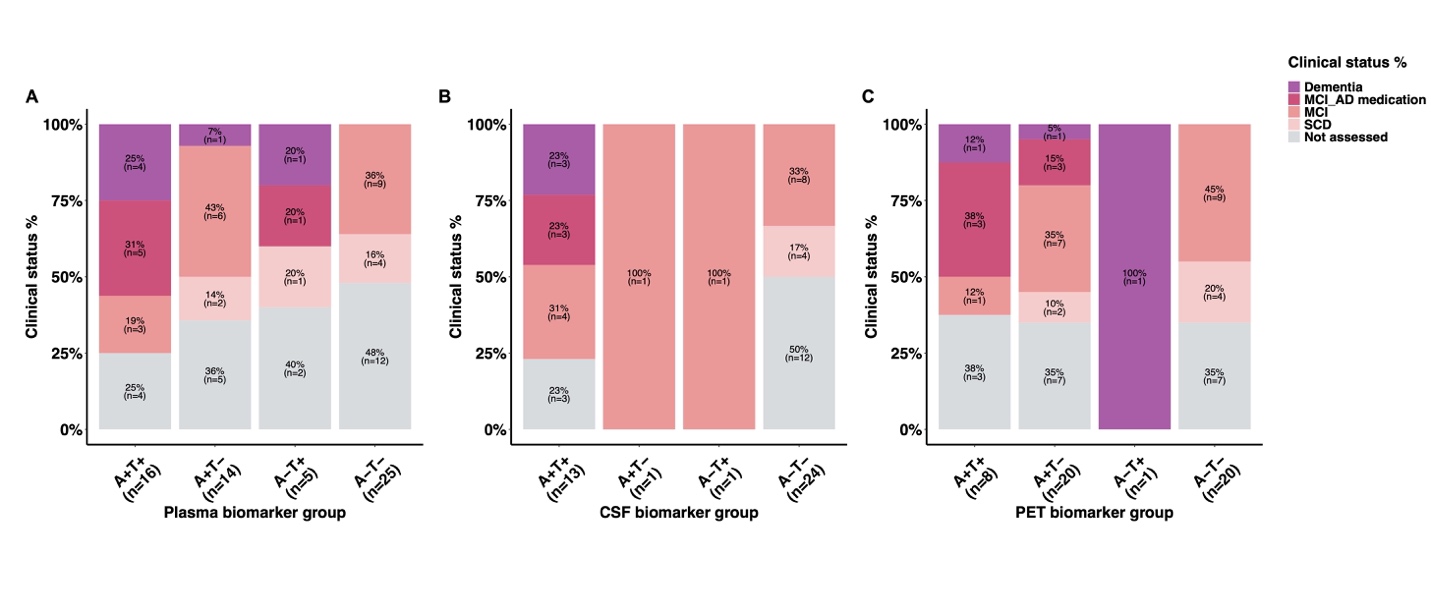


**Figure S3. Severity of the cognitive impairment based on clinical setting evaluations unblind to biomarker status.**

After their MCI classification most participants (38/62) were referred and followed by a physician in a clinical setting unblind to the biomarker classifications. Bar graphs represent the information available from the clinical settings for the **A)** plasma; **B)** CSF; and **C)** PET biomarker groups. Dementia = Participants diagnosed with dementia in the memory clinic without detailed information on the etiology. MCI = Mild cognitive impairment due to AD. MCI_AD medication = Participants diagnosed as MCI who are either enrolled in clinical trials or receiving AD medication. SCD = Subjective cognitive impairment. Not assessed = Participants who declined follow-up assessments at the memory clinic.


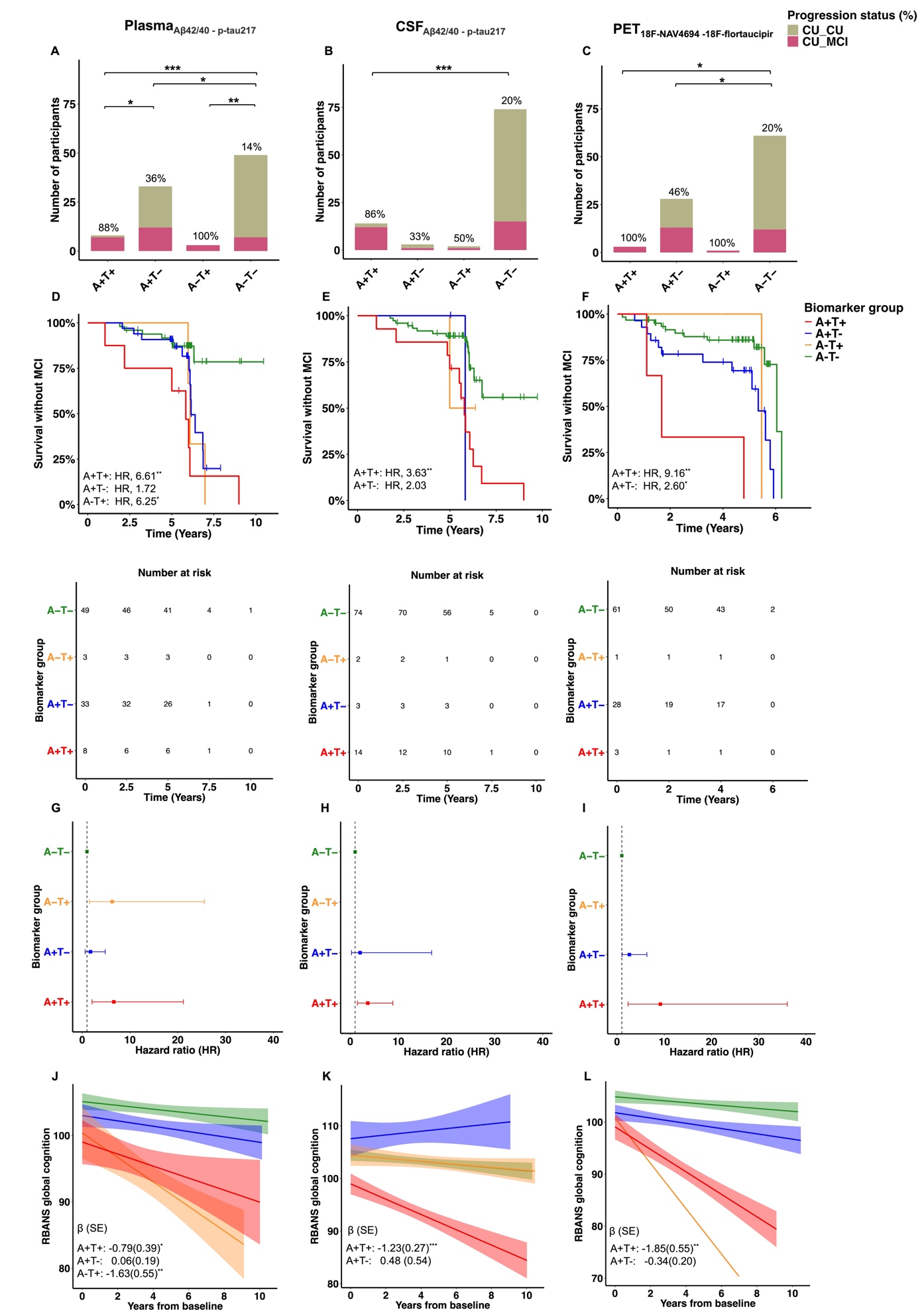


**Figure S4. Clinical progression to MCI in the subsample of 93 individuals with all AT biomarkers. A-C)** Bar graphs represent the proportion of participants who developed MCI in the subsample of 93 participants with all biomarker measurements across **A)** plasma Aβ_42/40_ and p-tau217; **B)** CSF Aβ_42/40_ and p-tau217; and **C)** PET biomarker profiles measured with ^18^F-NAV4694 and ^18^F-flortaucipir. **D-F)**. Survival curves reflecting the progression to MCI across **D)** plasma, **E)** CSF and **F)** PET biomarker groups. The vertical ticks on the curves refer to the censored participants, i.e., the loss of follow-up of the individuals. **G-I)** Forest plots showing HR and 95% confidence intervals from the survival analyses. **J-L)** Linear mixed effects models show the total cognitive score of RBANS over time across **J)** plasma, **K)** CSF and **L)** PET biomarker profiles. The linear mixed effects models analyses included annual cognitive data before and following plasma, CSF, and PET measures. Models included age at biomarker measurement, sex, and years of education as covariates. *Notes*: CU_CU = cognitively unimpaired older adults at the time of the biomarker measurement and remained cognitively unimpaired during follow-up; CU_MCI = cognitively unimpaired older adults at the time of the biomarker measurement, who progressed to mild cognitive impairment during follow-up. The A-T- group was used as reference. The A-T+ in the PET (n = 1) and CSF (n = 2) biomarker group is displayed for visualization purposes but was not included in the statistical analyses*.* HR = hazard ratios*;* ^*^ *P* < 0.05*, ^**^ P* < 0.01*, ^***^ P* < 0.001*.*

**
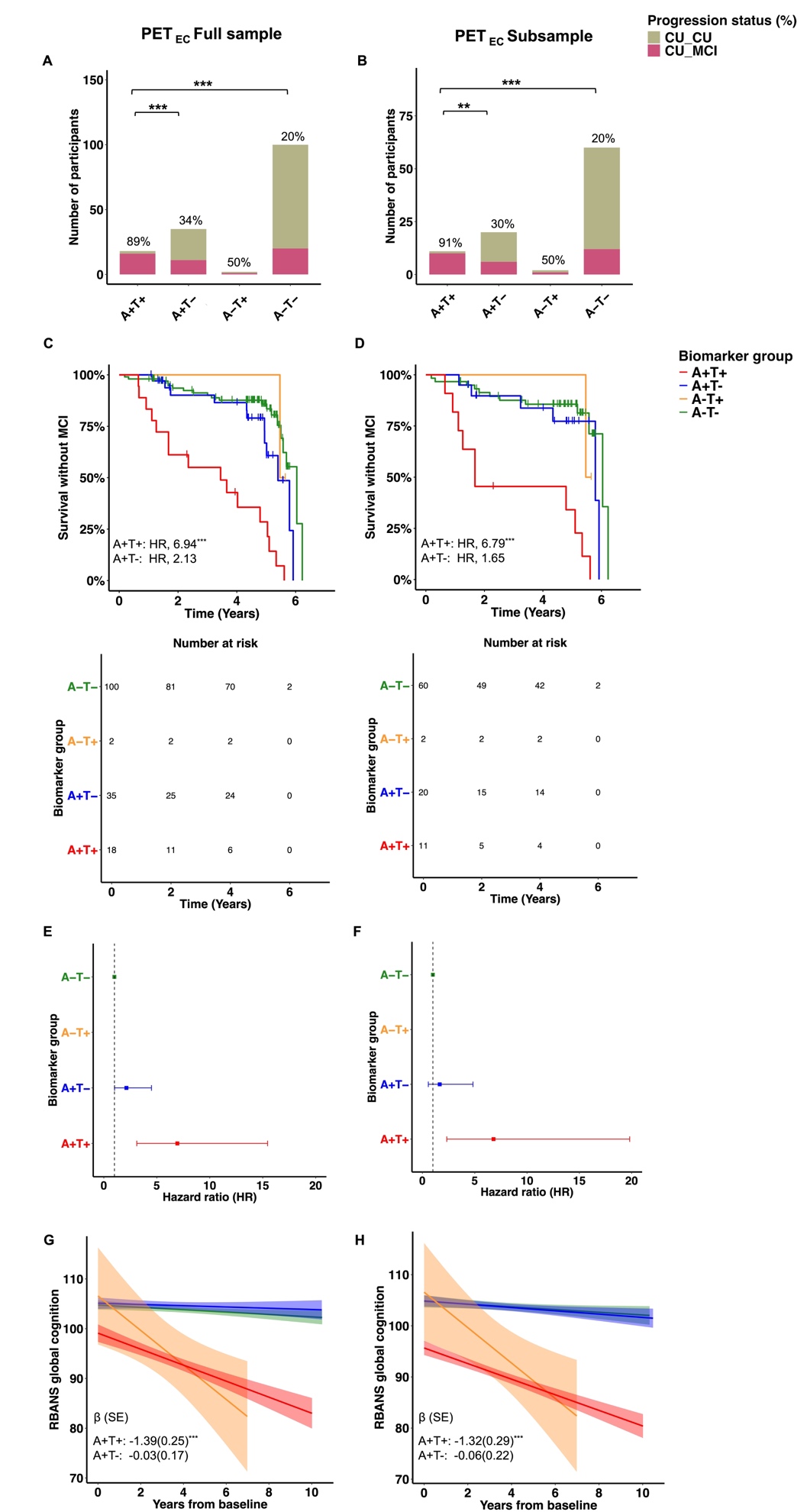
**

**Figure S5. Clinical progression to MCI across PET AT biomarker groups in the full sample and subsample using entorhinal cortex to define tau positivity. A-B)** Bar graphs represent the proportion of participants who developed MCI in the full sample and the subsample of 93 participants across PET biomarker profiles using entorhinal cortex to define tau positivity**. C- D)** Survival curves reflecting the progression to MCI in **C).** full sample, **D)**. subsample of participants with all biomarker measurements. The vertical ticks on the curves refer to censored participants, i.e., the loss of follow-up of the individuals. **E-F)** Forest plots showing HR and 95% confidence intervals from the survival analyses. **G-H)** Linear mixed effects models show the total cognitive score of RBANS over time. The linear mixed effects models analyses included annual cognitive data before and following PET measures. Models included age at PET, sex, and years of education as covariates. *Notes*: CU_CU = cognitively unimpaired older adults at the time of the biomarker measurement and remained cognitively unimpaired during follow-up; CU_MCI = cognitively unimpaired older adults at the time of the biomarker measurement, who progressed to mild cognitive impairment during follow-up. The A-T- group was used as reference. The A-T+ group is displayed for visualization purposes but was not included in the statistical analyses*.* HR = hazard ratios*; ^**^ P* < 0.01*, ^***^ P* < 0.001*.*

**
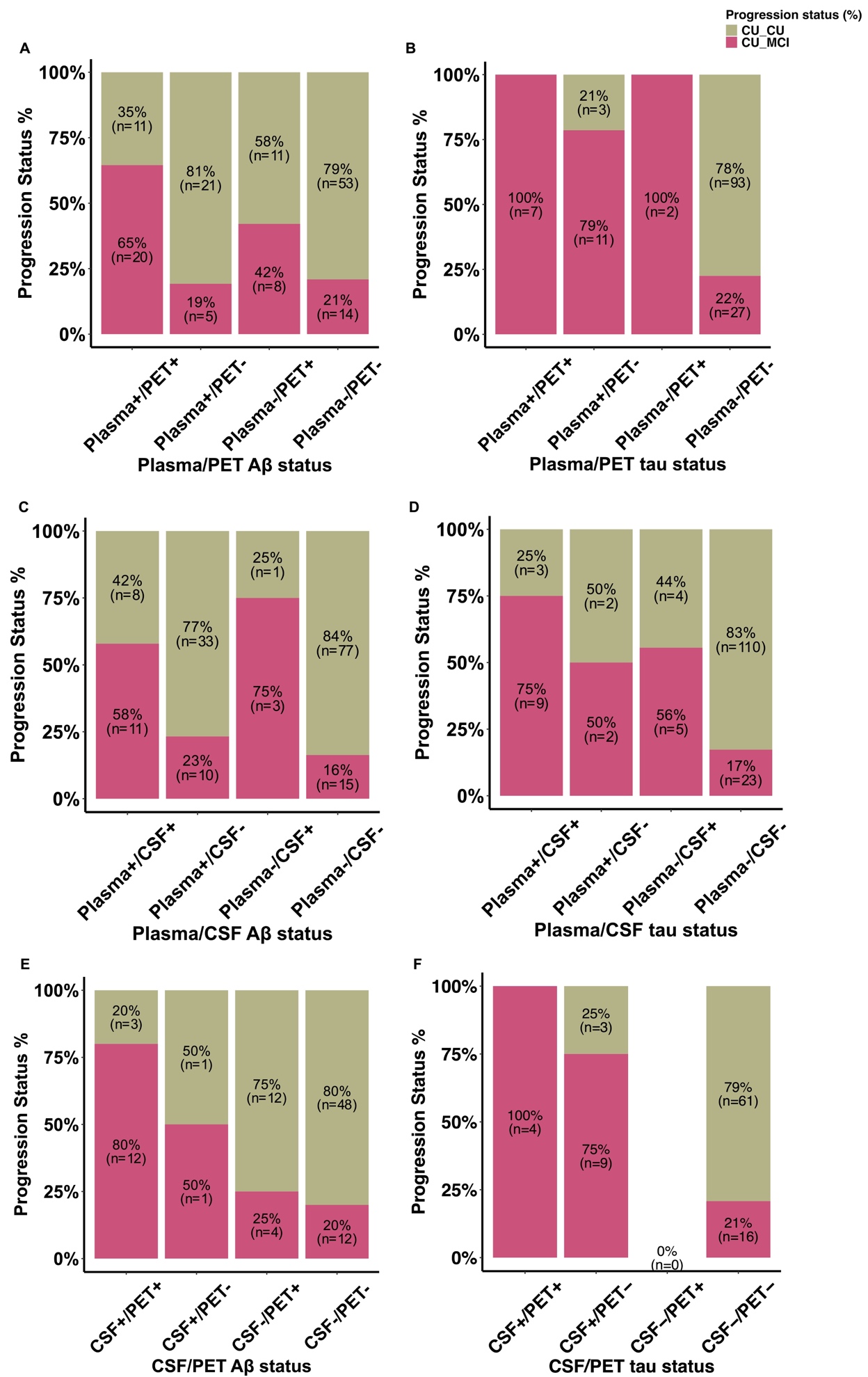
**

**Figure S6. Percentage of cognitively unimpaired vs MCI individuals between plasma, CSF and PET Aβ and tau biomarkers.** Bar graphs showing the percentage of participants staying CU (CU_CU) vs the one who progressed to MCI (CU_MCI) across **A)** plasma Aβ_42/40_ vs Aβ-PET biomarkers; **B)** Plasma p-tau217 vs meta-ROI tau-PET biomarkers; **C)** plasma Aβ_42/40_ vs CSF Aβ_42/40_ biomarkers; **D)** plasma p-tau217 vs CSF p-tau217 biomarkers; **E).** CSF Aβ_42/40_ vs Aβ-PET biomarkers; **F)** CSF p-tau217 vs meta-ROI tau-PET biomarkers. Colors indicate the cognitive progression status of participants. *Notes*: CU_CU = cognitively unimpaired older adults at the time of the biomarker measurement and remained cognitively unimpaired during follow-up; CU_MCI = cognitively unimpaired older adults at the time of the biomarker measurement, who progressed to mild cognitive impairment during follow-up. Cutoff values were 0.09 for plasma Aβ_42/40_; 0.072 for CSF Aβ_42/40_; 1.26 SUVR for Aβ-PET; 3.98 pg/mL for plasma p-tau217; 400.19 pg/mL for CSF p-tau217; and 1.29 SUVR for temporal meta-ROI tau-PET. n = 143 participants had both plasma and PET measurements, n = 158 had both plasma and CSF measurements, and n = 93 had both CSF and PET measurements.
